# Exoerythrocytic *Plasmodium* Parasites Secrete a Cysteine Protease Inhibitor Involved in Sporozoite Invasion and Capable of Blocking Cell Death of Host Hepatocytes

**DOI:** 10.1371/journal.ppat.1000825

**Published:** 2010-03-26

**Authors:** Annika Rennenberg, Christine Lehmann, Anna Heitmann, Tina Witt, Guido Hansen, Krishna Nagarajan, Christina Deschermeier, Vito Turk, Rolf Hilgenfeld, Volker T. Heussler

**Affiliations:** 1 Bernhard Nocht Institute for Tropical Medicine, Department of Molecular Parasitology, Hamburg, Germany; 2 Institute of Biochemistry, Center for Structural and Cell Biology in Medicine, University of Lübeck, Lübeck, Germany; 3 Josef Stefan Institute, Department of Biochemistry, Molecular and Structural Biology, Ljubljana, Slovenia; Case Western Reserve University, United States of America

## Abstract

*Plasmodium* parasites must control cysteine protease activity that is critical for hepatocyte invasion by sporozoites, liver stage development, host cell survival and merozoite liberation. Here we show that exoerythrocytic *P. berghei* parasites express a potent cysteine protease inhibitor (PbICP, *P. berghei* inhibitor of cysteine proteases). We provide evidence that it has an important function in sporozoite invasion and is capable of blocking hepatocyte cell death. Pre-incubation with specific anti-PbICP antiserum significantly decreased the ability of sporozoites to infect hepatocytes and expression of PbICP in mammalian cells protects them against peroxide- and camptothecin-induced cell death. PbICP is secreted by sporozoites prior to and after hepatocyte invasion, localizes to the parasitophorous vacuole as well as to the parasite cytoplasm in the schizont stage and is released into the host cell cytoplasm at the end of the liver stage. Like its homolog falstatin/PfICP in *P. falciparum*, PbICP consists of a classical N-terminal signal peptide, a long N-terminal extension region and a chagasin-like C-terminal domain. In exoerythrocytic parasites, PbICP is posttranslationally processed, leading to liberation of the C-terminal chagasin-like domain. Biochemical analysis has revealed that both full-length PbICP and the truncated C-terminal domain are very potent inhibitors of cathepsin L-like host and parasite cysteine proteases. The results presented in this study suggest that the inhibitor plays an important role in sporozoite invasion of host cells and in parasite survival during liver stage development by inhibiting host cell proteases involved in programmed cell death.

## Author Summary

*Plasmodium* sporozoites are transmitted by *Anopheles* mosquitoes to the vertebrate host. They migrate through the skin before entering blood vessels and being transported with the bloodstream to liver sinusoids. There the sporozoites transmigrate through Kupffer cells and several hepatocytes before they invade a final hepatocyte and develop into thousands of merozoites. These daughter parasites are transported inside host cell-derived vesicles (merosomes) back to the bloodstream where they are finally released and infect red blood cells. Most of these processes depend on the activity of proteases, which must be tightly controlled to avoid proteolytic destruction of the parasite. We have identified a potent cysteine protease inhibitor of the rodent parasite *Plasmodium berghei,* which is expressed throughout the life cycle of the parasite. The inhibitor appears to play a role in sporozoite invasion of host cells and in parasite survival during liver stage development by inhibiting host cell proteases involved in programmed cell death.

## Introduction

Malaria is caused by apicomplexan parasites of the genus *Plasmodium.* The infection of the vertebrate host begins with the inoculation of sporozoites into the dermis during blood feeding of an infected *Anopheles* mosquito [1],[2],[3],[4],[5],[6],[7]. Sporozoites traverse through different cell types [8],[9],[10],[11],[12] until they reach the liver via the bloodstream and finally invade hepatocytes. Here within 2 to 16 days, depending on the *Plasmodium* species, they develop inside a parasitophorous vacuole to several thousand red blood cell-infective merozoites [13]. Using the rodent malaria model parasite *P. berghei*, it was shown that infected hepatocytes are protected against apoptosis throughout liver stage development [14],[15],[16]. However, at the end of the liver stage, the parasite induces an unusual form of programmed cell death that facilitates the merozoites leaving the liver and gaining access to the bloodstream [16],[17]. Even during this complex liberation process, which includes parasite-dependent host cell death, classical host cell apoptosis is not induced and cell membrane integrity is maintained [16], suggesting that parasite molecules responsible for inhibition of apoptosis are translocated to the host cell cytoplasm, at least during this last phase of parasite development in hepatocytes.

As in the blood stage and mosquito stage [18],[19], it was shown that cysteine proteases play a crucial role in the infection of the liver by *Plasmodium* sporozoites [20],[21], in parasite development in liver cells and in the liberation of the fully developed liver merozoites [16]. Therefore, a tight regulation of protease activity is critical for the survival of the parasite throughout its life cycle. Additionally, intra- and extracellular *Plasmodium* parasites are exposed to host cell proteases and it is likely that they have evolved mechanisms to counteract proteolytic digestion.

Host cell proteases are often involved in pathogen defense mechanisms and a number of other parasites have already been shown to express cysteine protease inhibitors that block these proteases. These include host cell proteases involved in antigen presentation, cytokine responses and host cell apoptosis and proteases that are stored in potentially fusogenic organelles like lysosomes and are liberated upon pathogen recognition [22],[23],[24],[25],[26],[27],[28],[29],[30],[31],[32],[33],[34].

Short-term regulation of parasite as well as host proteases can be mediated by specific parasite-derived inhibitors. A prominent example is chagasin, which is expressed by *Trypanosoma cruzi* and was the first identified member of a new superfamily of reversible, tight-binding cysteine protease inhibitors [35]. Structurally similar inhibitors were found in *Trypanosoma brucei, Pseudomonas aeruginosa, Leishmania mexicana, Leishmania major, Entamoeba histolytica*, *P. falciparum* and *Toxoplasma gondii* [36],[37],[38],[39],[40],[41],[42],[43]. Chagasin-like inhibitors (also termed ICPs for inhibitor of cysteine proteases) are suggested to regulate both endogenous parasite-derived cysteine proteases (*T. brucei, T. cruzi, P. falciparum, E. histolytica*) and/or host cell proteases (*L. mexicana, P. aeruginosa, P. falciparum*) [35],[37],[38],[39],[40],[41],[42],[44],[45],[46].

The *P. falciparum* ICP (PfICP), termed falstatin, has been described previously for the blood stage of the human malaria parasite [40]. Falstatin/PfICP has been characterized as a potent inhibitor of various parasite and host cell cysteine proteases and is expressed by blood schizonts, merozoites and rings but not in trophozoites. Incubation of late schizonts with neutralizing antibodies against falstatin/PfICP partially blocked subsequent invasion of erythrocytes by merozoites, suggesting that regulation of cysteine protease activity is important for this process.

Here we report on the falstatin homolog of the rodent malaria parasite *P. berghei,* which we name PbICP for *Plasmodium berghei* inhibitor of cysteine proteases, following the common nomenclature for the entire inhibitor family. PbICP appears to play a critical role at least during the parasite liver and blood stages in the vertebrate host. We analyzed specifically the exoerythrocytic parasite stages and suggest a function of PbICP in sporozoite invasion and host cell survival.

## Materials and Methods

### Experimental animals

Animals were obtained from Charles River Laboratories. All animal work was conducted in compliance with regulations created and approved by the ethical committee of Hamburg state authorities (Nr. FI 28/06).

### Sequence alignments and expression and purification of recombinant PbICP proteins

Homology searches, multiple alignments and secondary structure predictions were performed with public BLAST search tools (PlasmoDB), the Clustal W program (EMBL-EBI) and structure prediction programs (PredictProtein, Jpred).

The *pbicp* gene (full length, PlasmoDB: PB000502.02.0), *pbicp-n* and *pbicp-c* were amplified from cDNA of *P. berghei* ANKA wildtype mixed blood stage parasites using the following primer pairs: PbICP-fw (5′-GG GAATTCGAAGATAACGACATATACTCTTTTGATATC-3′) / PbICP-rv (5′- CCCAAGCTT TTATTGGACAGTCACGTATATAAT-3′) to clone MBP-PbICP full length, PbICP-C-fw1 (5′-TTGAATTCGGAGATGAAAAATGTGGTAAATCA-3′) / PbICP-C-rv1 (5′-TTGGATCC TTATTGGACAGTCACGTATATAAT-3′) to clone MBP-PbICP-C, PbICP-C-fw2 (5′- TTCATATG GGAGATGAAAAATGTGGTAAATCA-3′) / PbICP-C-rv2 (5′-TTGAATTC TTATTGGACAGTCACGTATATAAT-3′) to clone His-PbICP-C and PbICP-C without tag, PbICP-N-fw1 (5′-GGGAATTCGAAGATAACGACATATACTCTTTTGATATC-3′) / PbICP-N-rv1 (5′-TT GGATCC TGGTTAAATGAGTTGTATGAAGTAGTTGGG -3′) to clone MBP-PbICP-N for antibody production, PbICP-N-fw2 (5′-GGGAATTCGAAGATAACGACATATACTCTTTTGATATC-3′) / PbICP-N-rv2 (5′-TTGGATCC AGTCAATTCATATTTACTATCAACTTTACCA -3′) to clone MBP-PbICP-N for protease assays.

The PCR products were cloned into the bacterial expression vectors (pJC25 for untagged PbICP-C, derivative of pJC20 [47]; pJC45 for N-terminal His_10_-tagged PbICP-C [48]; pMAL-cRI for N-terminal MBP tagged full length PbICP, PbICP-C and PbICP-N) using the appropriate restriction enzymes (New England Biolabs) and clones were analyzed by sequencing. Untagged PbICP-C and His-PbICP-C were expressed in the BL21 (DE3) pAPlacI *E. coli* strain [48] and purified using a MonoQ column (Pharmacia) or Ni-NTA resin (Qiagen), respectively. MBP-PbICP (full length), MBP-PbICP-C and MBP-PbICP-N were expressed in the BL21 *E. coli* strain (Stratagene) and purified using amylose resin (New England Biolabs). The MBP-tag of PbICP (full length) was removed by factor Xa (New England Biolabs) and untagged PbICP was purified by using a MonoQ column (Pharmacia).

### Protease assays

Cathepsin L [49], papain (Sigma) or falcipain-2 [50] were incubated with 600 µM Z-Phe-Arg-pNA substrate (Bachem) in the presence or absence of indicated amounts of the recombinant purified proteins. Assay buffers were 100 mM acetate buffer, 1 mM DTT, pH 5.0 for cathepsin L and 100 mM acetate buffer, 10 mM DTT, pH 5.5 for papain and falcipain-2. Cathepsin B (Sigma) was incubated with 600 µM Z-Arg-Arg-pNA substrate (Bachem) in the presence or absence of the indicated amounts of the recombinant purified proteins in 100 mM KH_2_PO_4_, 2 mM EDTA, 10 mM DTT, pH 6.0. Photometric product formation (E) was measured every 10 seconds and activity was calculated from the linear part of the graph (ΔE/Δt). Protease activity in the presence of the control protein MBP was set to 100% and residual activity in the presence of recombinant PbICP constructs was calculated.

### Duplex-PCR

Total RNA was isolated from 10^6^ wildtype oocyst sporozoites, 6×10^5^ wildtype salivary gland sporozoites, infected HepG2 cells at different time points after infection or from saponin-treated (0,05%, Sigma) blood stage parasites using the RNA Extract Kit II (Macherey and Nagel). First strand cDNA synthesis was performed with the Superscript™ First-Strand Synthesis System for RT-PCR (Invitrogen).

Duplex-PCR was performed with the two primer pairs PbICP-fw (5′-ATGCTCCATCCTAGCCCTTT-3′) / PbICP-rev (5′-CCACTTTCATTCATTGTGTTGTT-3′) and Pbtubulin-fw (5′- TGGAGCAGGAAATAACTGGG-3′) / Pbtubulin-rev (5′-ACCTGACATAGCGGCTGAAA-3′). All RNA preparations were free of genomic DNA (gDNA) contamination as no PCR product was obtained when reverse transcriptase had been omitted from the RT-PCR (negative control).

### Generation of antibodies

25 µg of each of the purified fusion proteins His-PbICP-C, MBP-PbICP (full length) and MBP-PbICP-N in PBS buffer were mixed with one volume Freund's adjuvant complete (Sigma) and intraperitoneally injected into BALB/c or NMRI mice. After two weeks, the mice were boosted with the same amount of protein mixed with Freund's adjuvant incomplete (Sigma) followed by a second boost two weeks later. Immunized mice were killed after confirming the antibody response in preliminary experiments, and blood was collected by cardiac puncture. Antisera were obtained after centrifugation and diluted with one volume glycerol (Roth) for long term storage at −20°C.

Rabbit antisera against His-PbICP-C, rabbit antisera against a peptide within the PbICP-N sequence and the appropriate preimmune sera were obtained from Eurogentec S.A.

### *In vitro* infection of HepG2 cells

Human hepatoma cells (HepG2) were obtained from the European cell culture collection. Cells were cultivated at 37°C and 5% CO_2_ in EMEM (PAA) containing 10% fetal calf serum, 2 mM L-glutamine, 100 U/ml penicillin, 100 µg/ml streptomycin (all PAA Laboratories GmbH). For infection, 5×10^4^ cells were seeded per coverslip in a 24-well plate. *P. berghei* (ANKA) sporozoites were prepared from salivary glands of infected *Anopheles stephensi* mosquitoes and incubated with HepG2 cells in EMEM (PAA) containing 3% bovine serum albumin (Sigma), 2 mM L-glutamine, 100 U/ml penicillin, 100 µg/ml streptomycin (all PAA Laboratories GmbH) at 37°C and 5% CO_2_. After 2 hours, cells were washed and incubated in fresh culture medium for the indicated times.

### SDS-PAGE and Western blotting

Parasite proteins were separated on 12% to 14% SDS-PAGE gels under reducing conditions and transferred to nitrocellulose membranes. Membranes were probed with rabbit antisera directed against His-PbICP-C or a peptide of PbICP-N, anti-GFP mouse monoclonal antibodies (Roche) or rabbit antisera (Molecular Probes) and mouse anti-tubulin monoclonal antibody (Sigma). Horseradish peroxidase-conjugated anti-rabbit (Cell Signalling) or anti-mouse IgG (Pierce or Rockland) and enhancing chemiluminescence substrate detection kits (Pierce and Bio-Rad) were used for detection.

### Immunofluorescence microscopy

#### Sporozoite IFA

Sporozoites were isolated from salivary glands of *A. stephensi* mosquitoes and incubated on glass coverslips with or without HepG2 cells in EMEM (PAA) containing 3% bovine serum albumin (Sigma), 2 mM L-glutamine, 100 U/ml penicillin and 100 µg/ml streptomycin (all PAA Laboratories GmbH) at 37°C and 5% CO_2_. After 2 hours, medium was carefully removed and sporozoites were fixed with 4% formaldehyde in PBS (20 min, room temperature), permeabilized with ice-cold methanol (10 min) and incubated with primary antisera (rabbit anti-His-PbICP-C, mouse anti-CSP, mouse anti-TRAP) and subsequently with fluorescently labeled secondary antibodies (Cy2-labeled antibodies, Dianova and Alexa594-labeled antibodies, Molecular Probes). DNA was visualized by staining with 10 µg/ml DAPI (Sigma). Labeled cells were analyzed by widefield or confocal microscopy using the Leica DM RB or Olympus FV1000.

#### Staining of unfixed sporozoites

Sporozoites expressing cytosolic mCherry [51] were isolated from salivary glands of *A. stephensi* mosquitoes and incubated for 40 minutes on ice in PBS with a 1∶100 dilution of rabbit antiserum directed against His-PbICP-C, the appropriate preimmune serum or mouse antiserum directed against CSP. Sporozoites were washed twice with cold PBS and subsequently incubated with a 1∶200 dilution of the Cy2-labeled anti-rabbit or anti-mouse secondary antibody (Dianova) and Hoechst 33258 (Molecular Probes) for 30 min on ice. Sporozoites were washed twice, settled on coverslips with DAKO Fluorescent Mounting Medium (DAKO) and immediately analyzed by widefield fluorescence microscopy using the Leica DM RB.

#### IFA of infected HepG2 cells

HepG2 cells were infected as described above. After the indicated time periods, cells were fixed with 4% formaldehyde in PBS (20 min, room temperature), permeabilized with ice-cold methanol (10 min) and incubated with primary antibody (chicken anti-ExpI, mouse anti-CSP) and subsequently with fluorescently labeled secondary antibodies (Cy2-labeled antibodies, Dianova and Alexa594-labeled antibodies, Molecular Probes). DNA was visualized by staining with 10 µg/ml DAPI (Sigma). Labeled cells were analyzed by widefield or confocal microscopy using the Leica DM RB or Olympus FV1000.

### Immunoelectron microscopy

Midguts and salivary glands of *P. berghei* infected *A. stephensi* mosquitoes were isolated, fixed in 2% paraformaldehyde and 0.025% glutaraldehyde and slowly embedded in LR WhiteTM Resin (London Resin Company) after dehydration. Briefly, ultrathin sections were incubated with rabbit antiserum directed against His-PbICP-C and subsequently with gold (10 nm)-labeled Protein A.

For double staining, ultrathin sections were first incubated with anti-TRAP mouse antiserum and subsequently with gold (10 nm)-labeled anti-mouse IgG and afterwards with rabbit antiserum directed against His-PbICP-C and with gold (25 nm)-labeled Protein A.

Sections were stained with 2% uranyl acetate and 1∶10 diluted Reynold's lead citrate solution in 0.01 N NaOH. Sections were analyzed using the FEI Tecnai TEM.

### Transfection of *P. berghei*

DNA was amplified from cDNA of *P. berghei* ANKA wildtype mixed blood stage parasites using the primer pair PbICP^pL17^-fw (5′-CTG*GGATCC*ATGAAAAGTATAACTTTTTTCGTGTTTAAT-3′) / PbICP^pL17^-rev (5′-CTG*GGATCC*TTGGACAGTCACGTATATAATTTTAGTGTT-3′). The resulting fragment was cloned into the *P. berghei* GFP transfection plasmid pL0017 (MR4) using the *Bam*HI site in front of the *gfp* gene. The plasmid was linearized for transfection using the restriction sites of *Sac*II and *Apa*I in the ssu integration site sequence. Schizont-stage parasites were transfected with 2.5 and 7.5 µg purified plasmid DNA as described by Janse et al. [52].

#### Analysis of the transgenic parasites during the liver stage

To monitor parasite growth over the course of development, parasite size was measured by the density slice module of the OpenLab software version 5.0.2. Briefly, at different time points after infection (24 hpi (hours post infection), 48 hpi, 63 hpi), transgenic parasites over-expressing PbICP-GFP and control parasites were photographed. Images from each time point were merged and OpenLab software version 5.0.2 was used to produce a density slice of the image and to calculate the parasite areas.

An inside/outside assay to investigate infection efficiency was performed as follows: 1×10^5^ HepG2 cells were seeded per coverslip in a 24-well plate. The following day, transgenic PbICP-GFP sporozoites and GFPcon sporozoites as control were prepared from salivary glands of infected *A. stephensi* mosquitoes and counted in a Neubauer chamber. 2×10^4^ sporozoites per sample were added to the HepG2 cells. After 1 h incubation at 37°C and 5% CO_2_, cells were fixed for 2 min with 2% formaldehyde in PBS (no permeabilization) and incubated with rabbit or mouse anti-CSP antiserum and subsequently with fluorescently labeled secondary antibody (Alexa594-labeled antibodies, Dianova). DNA was visualized by staining with 10 µg/ml DAPI (Sigma). Sporozoites that have invaded cells are protected against CSP staining and are visualized just by the GFP fluorescence. In contrast, free GFP-positive sporozoites are stained by the CSP antibodies. Free and intracellular sporozoites were counted and their percentages were calculated.

### Sporozoites neutralization assays

#### Pre-incubation in antisera

The neutralization assay was mainly performed as described by Kumar et al. [53]. 1×10^5^ HepG2 cells were seeded per coverslip in a 24-well plate. The following day, *P. berghei* (ANKA) sporozoites were prepared from salivary glands of infected *A. stephensi* mosquitoes and counted in a Neubauer chamber. For the inside/outside assay, red fluorescent sporozoites expressing cytosolic mCherry protein were used [51]. 2×10^4^ sporozoites per sample were incubated in 30 µl of the anti-His-PbICP-C rabbit antiserum, the appropriate preimmune serum or anti-CSP rabbit antiserum for 40 min on ice. Afterwards, pre-warmed medium containing 3% bovine serum albumin (Sigma), 2 mM L-glutamine, 100 U/ml penicillin and 100 µg/ml streptomycin (all PAA Laboratories GmbH) was added to the samples. The sporozoite suspension was then added to HepG2 cells. Analysis of the neutralization assays was performed by different established methods (transmigration assay, inside/outside assay for invading sporozoites or counting of infected hepatocytes 30 hpi (detailed description see below)).

#### Transmigration assay

The suspension of sporozoites was mixed with 1 mg/ml dextran-fluorescein (10,000 MW, Molecular Probes) prior being added to the HepG2 cells. 1 h after incubation at 37°C and 5% CO_2_, cells were washed three times in PBS, fixed with 4% formaldehyde in PBS (20 min, room temperature) and permeabilized with ice-cold methanol (10 minutes). To visualize the sporozoites, CSP-staining was performed (mouse anti-CSP, Alexa594-labeled anti-mouse antibody, Molecular Probes). DNA was stained with 10 µg/ml DAPI (Sigma). The number of transmigrated cells was determined by calculating the percentage of dextran-fluorescein-positive cells as a percentage of all cells. HepG2 cells without sporozoites served as control (basal rate of wounded cells).

#### Inside/outside assay

1 h after incubation at 37°C and 5% CO_2_, cells were fixed for 2 min with 2% formaldehyde in PBS (no permeabilization) and incubated with rabbit or mouse anti-CSP antiserum and subsequently with fluorescently labeled secondary antibody (Cy2-labeled antibodies, Dianova). DNA was visualized by staining with 10 µg/ml DAPI (Sigma). Sporozoites that have invaded cells are protected against the CSP-staining and were visualized only by the mCherry expression. Free mCherry sporozoites were additionally stained by the CSP antibodies. Sporozoites were counted and the percentage of free and intracellular sporozoites was calculated.

#### Counting of infected hepatocytes in the schizont stage

After 2 h incubation at 37°C and 5% CO_2_, cells were washed and fresh culture medium was added. 24 to 30 hours after infection, cells were used for IFA and the numbers of infected HepG2 cells per coverslip were determined. More than 200 infected cells were counted in the preimmune controls and set to 100% for each of the three independent experiments.

### Transfection of HepG2 cells and cell death assays

DNA was amplified from cDNA of *P. berghei* ANKA wildtype mixed blood stage parasites using the primer pair PbICP-C^pEGFP^-fw (5′-TTGAATTCGGAGATGAAAAATGTGGTAAATCA-3′) / PbICP-C ^pEGFP^ -rev (5′-TTGGATCCTTATTGGACAGTCACGTATATAAT-3′).

The resulting fragment was cloned into the pEGFP-C2 vector (Clontech) to generate a GFP-PbICP-C-fusion protein. The vector pEGFP-C2 alone was used as the GFP control.

HepG2 cells were grown in single wells of a 6-well plate at 70–80% confluence and transfected using MATra technology (IBA) as recommended by the manufacturer. Briefly, cells were washed with PBS and reduced-serum OptiMEM media (PAA) was added. 5 µg of the appropriate plasmid DNA was pre-incubated with 5 µl of MATra-A reagent in OptiMEM media for 20 min at room temperature and then added to the cells. The cells were incubated with the DNA-MATra complex on a universal magnetic plate (IBA) for 30 min at 37°C and 5% CO_2_.

The next day transfected cells were treated with 70 nM tBHP (tert-butylhydroperoxid, Sigma-Aldrich) for 4 hours or 1 µM CAM (camptothecin, Sigma-Aldrich) for 48 hours. Cells were washed with PBS and stained with 25 nM TMRE (tetramethylrhodaminethylesterperchlorat, Molecular Probes) and 16 µM Hoechst 33258 (Molecular Probes) at 37°C and 5% CO_2_ for 30 min. Live imaging was performed using an inverse microscope (Axiovert 25, Zeiss) and the Openlab 5 software (Improvision).

## Results

### Sequence peculiarities, expression and biochemical characterization of PbICP

To investigate the regulation of cysteine protease activity by exoerythrocytic *P. berghei* parasites, we analyzed the structural and biochemical properties of the *P. berghei* homolog of falstatin/PfICP, PbICP. It belongs to the chagasin-like inhibitor family (MEROPS-family I42, termed ICP for inhibitor of cysteine proteases). ICPs are so far the only cysteine protease inhibitors known to exist in protozoan parasites. Typically, ICPs are small proteins with a low sequence similarity to each other but a characteristic β-sheet-rich secondary structure similar to immunoglobulins [54],[55],[56],[57]. Three loops (L2, L4, L6) form a wedge-like structure that extends into the active site cleft of C1 family cysteine proteases (papain superfamily). The ICPs of the apicomplexan protozoan parasites *Plasmodium* and *Toxoplasma gondii* are unusual chagasin family members [40],[43] in that they differ more than other members in sequence and even in length of the wedge-forming loops (Figure S1, S2A). Moreover, the *Plasmodium* ICPs contain an additional N-terminal extension region resulting in an overall molecular weight of about 40 kDa. PbICP shares approximately 40% amino acid sequence identity with falstatin/PfICP but only about 21% with chagasin of *Trypanosoma cruzi*, the first described member of the inhibitor family. Secondary-structure prediction programs propose that PbICP contains the characteristic β-sheet-rich structure with two additional β-strands (β5′and β5″) between the elongated L3 and L4 (Figure S2B).

The *pbicp* gene consists of two exons (exon 1: 63 bp; exon 2: 999 bp) and an intron of 519 bp. The first exon codes for a classical signal peptide while the second exon codes for the mature protein.

**Figure 1:**
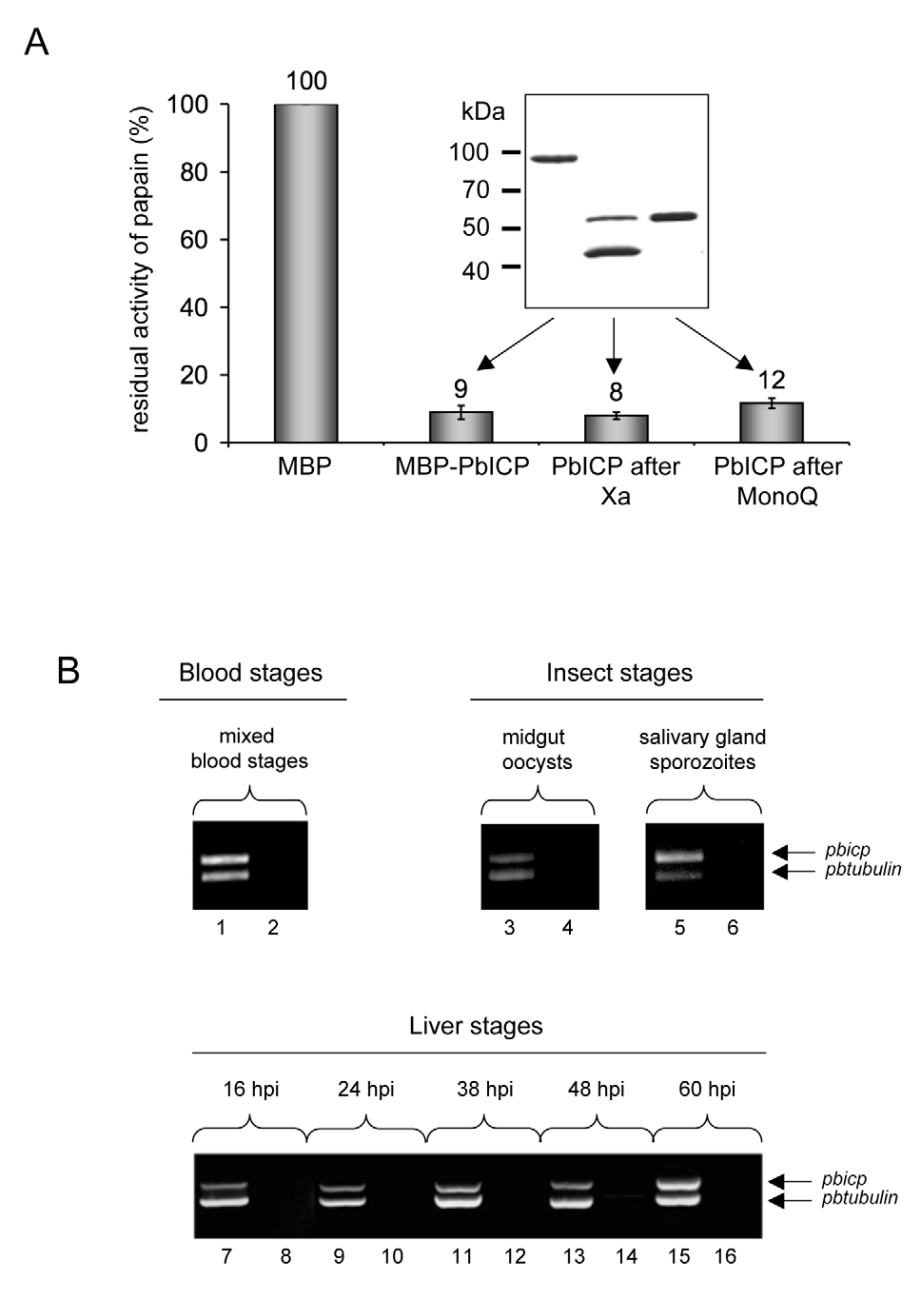
PbICP is a potent inhibitor of C1 cysteine proteases and the *pbicp* gene is constitutively transcribed. (**A**) Recombinant PbICP inhibits papain activity. Recombinant PbICP was produced in *E. coli* as an MBP-tagged soluble protein and purified from the bacterial lysate by amylose-bead affinity chromatography (insert, lane 1). The MBP tag was cleaved off by factor Xa digestion (insert, lane 2) and PbICP was purified using a MonoQ column (insert, lane 3). Proteins were resolved by SDS-PAGE and stained with Coomassie Blue. Hydrolysis of the substrate Z-Phe-Arg-pNA by papain was measured in the presence of 100 nM of the control protein MBP or 100 nM of MBP-PbICP, factor Xa-cleaved MBP-PbICP or PbICP after tag cleavage and MonoQ purification. Protease activity in presence of 100 nM MBP was considered 100% and the percentage of residual protease activity was calculated from that. MBP: maltose binding protein. (**B**) Constitutive transcription of the *pbicp* gene. RNA was isolated from *P. berghei*-infected mouse blood, mosquito midgut and salivary glands and from infected HepG2 cells. The purified RNA was then used for a duplex RT-PCR with specific primer pairs for the amplification of *pbicp* cDNA and the *tubulin* cDNA of *P. berghei*. In samples with odd numbers, cDNA was used as a template. In samples with even numbers, reverse transcriptase was omitted from the cDNA preparation reaction to control for the presence of genomic DNA.

To confirm that PbICP acts as a cysteine protease inhibitor, we produced recombinant PbICP as an MBP (maltose binding protein) fusion protein in *E. coli,* purified the protein by affinity chromatography and removed the MBP tag by factor Xa digestion and subsequent ion exchange chromatography (Figure 1A). PAGE analysis of the purified proteins revealed MBP-PbICP to be 97 kDa in size (55 kDa without tag), which is considerably larger than the size calculated from the amino acid sequence (82 kDa and 40 kDa with and without tag, respectively). We tested the purified MBP-tagged and untagged PbICP in protease assays using cysteine proteases of the C1 family that are the known targets of chagasin-like inhibitors. 1 µM and 100 nM of both tagged and untagged PbICP strongly inhibited papain, the *P. falciparum* cysteine protease falcipain-2 and human cathepsin-L (Figure 1A, Table 1). Like falstatin [40], PbICP did not inhibit cathepsin B. This is in contrast to chagasin, the ICP of *L. mexicana* (LmICP) and the ICP of *T. brucei* (TbICP) [37],[58], which are all reported to inhibit cathepsin B, to various extents. We compared the ICP1 of *E. histolytica* (EhICP1) and PbICP in their capacity to inhibit cathepsin B and found that EhICP1 blocked this protease but PbICP did not (data not shown).

**Table 1:** Recombinant PbICP is a potent inhibitor of cysteine proteases but not of cathepsin B.

	papain	cathepsin L	falcipain-2	cathepsin B
1 µM MBP	100	100	100	100
1 µM MBP-PbICP	**3±1**	**0±0,2**	**0±0,1**	**100±2**
100 nM MBP	100	100	100	100
100 nM MBP-PbICP	**9±2**	**1±1**	**n.d.**	**102±0,1**
100 nM PbICP	**12±2**	**16±13**	**24±5**	**103±2**

Substrate hydrolysis was measured in the presence of the control protein MBP (1 µM or 100 nM) or in the presence of MBP-PbICP (1 µM or 100 nM) or PbICP (100 nM). Experiments were performed in triplicate. Protease activity in the presence of 1 µM and 100 nM MBP, respectively was considered as 100% and the percentage of residual protease activity in the presence of the inhibitor was calculated. n.d. not done.

### PbICP is expressed throughout the life cycle of *P. berghei*

Our next aim was to analyze expression of PbICP in various life cycle stages of *P. berghei*. First, we investigated *pbicp* mRNA expression. RNA was isolated from *P. berghei* insect stages (oocyst and salivary gland sporozoites), red blood cells from infected NMRI mice and infected hepatoma cells. Duplex RT-PCR assays suggested that *pbicp*, like *tubulin*, is constitutively transcribed throughout the life cycle stages analyzed (Figure 1B).

To determine PbICP expression in the exoerythrocytic parasite stages at the protein level, we produced specific antisera against recombinant PbICP in rabbits as well as mice and used them in indirect immunofluorescence analysis (IFA), immunoelectron microscopy (IEM) and western blot analysis. An overview is provided of the different antisera generated against the full-length PbICP, PbICP domains (PbICP-N and PbICP-C) and PbICP peptides (Figure S3), as well as of representative experiments demonstrating the specificity of the antisera (Figure S4 and S5).

**Figure 2:**
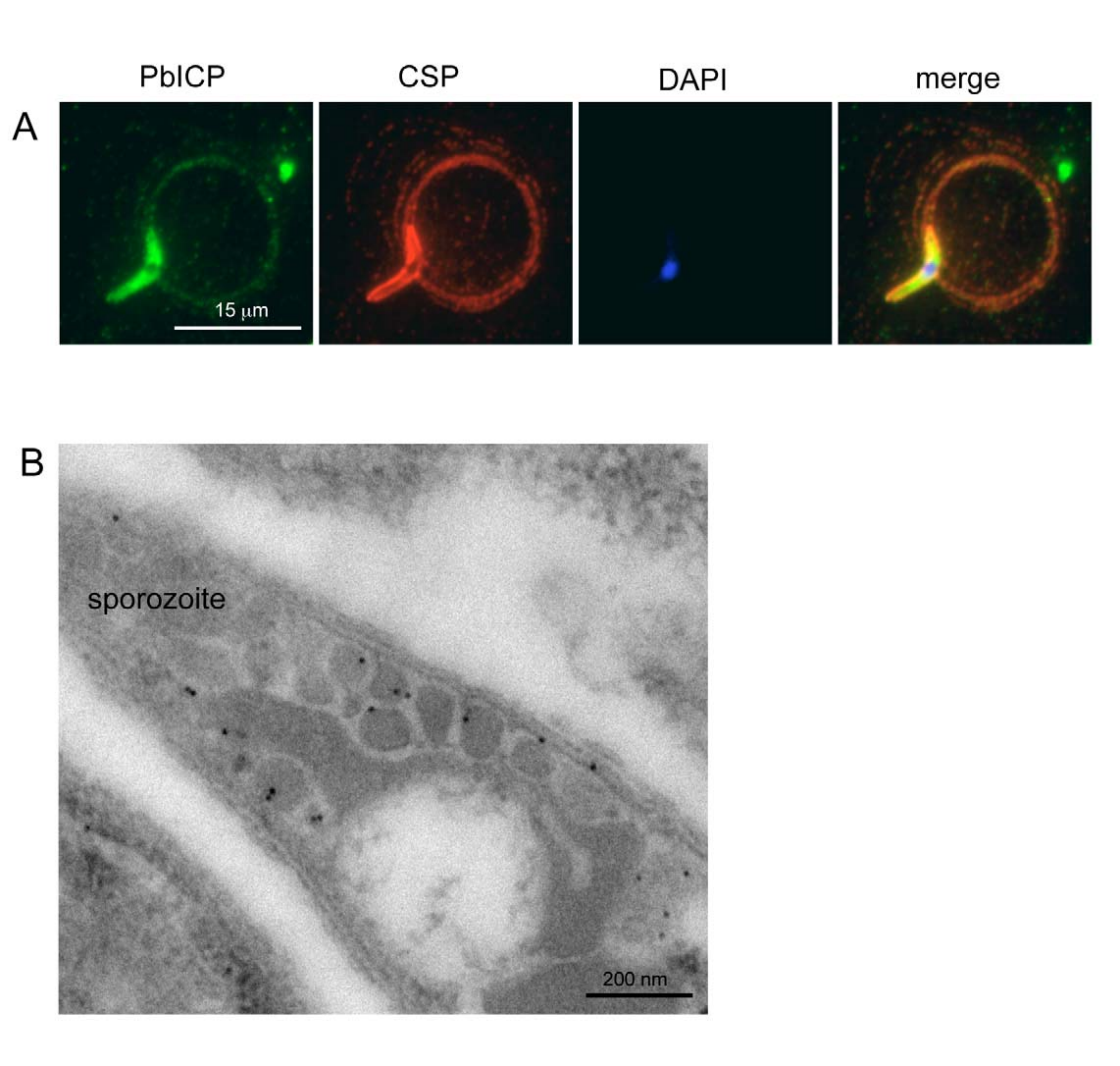
PbICP is secreted by salivary gland sporozoites. (**A**) Widefield IFA of *P. berghei* sporozoites that have been allowed to glide on glass coverslips. Sporozoites were fixed after 1 h and subsequently stained with anti-CSP antiserum (mouse, red) and polyclonal antiserum against PbICP-C (rabbit, green). DNA was stained with DAPI (blue). Like the shedded surface protein CSP, PbICP was detected in a patchy pattern in the trails of the circling sporozoites. (**B**) IEM confirms PbICP localization in vesicles of a midgut sporozoite. *P. berghei* infected *A. stephensi* midguts were fixed 20 days after infection and prepared for IEM. Ultrathin sections were stained with polyclonal antiserum directed against PbICP-C (rabbit) and subsequently with gold-labeled Protein A (10 nm gold particles). The sections were contrasted with uranyl acetate and lead citrate.

In agreement with the transcription profile of the *pbicp* gene, the PbICP protein could be detected in all exoerythrocytic parasite stages (Figures 2–4). PbICP expression and localization in sporozoites, liver schizonts and detached cells/merosomes will now be explained in detail.

**Figure 3 ppat-1000825-g003:**
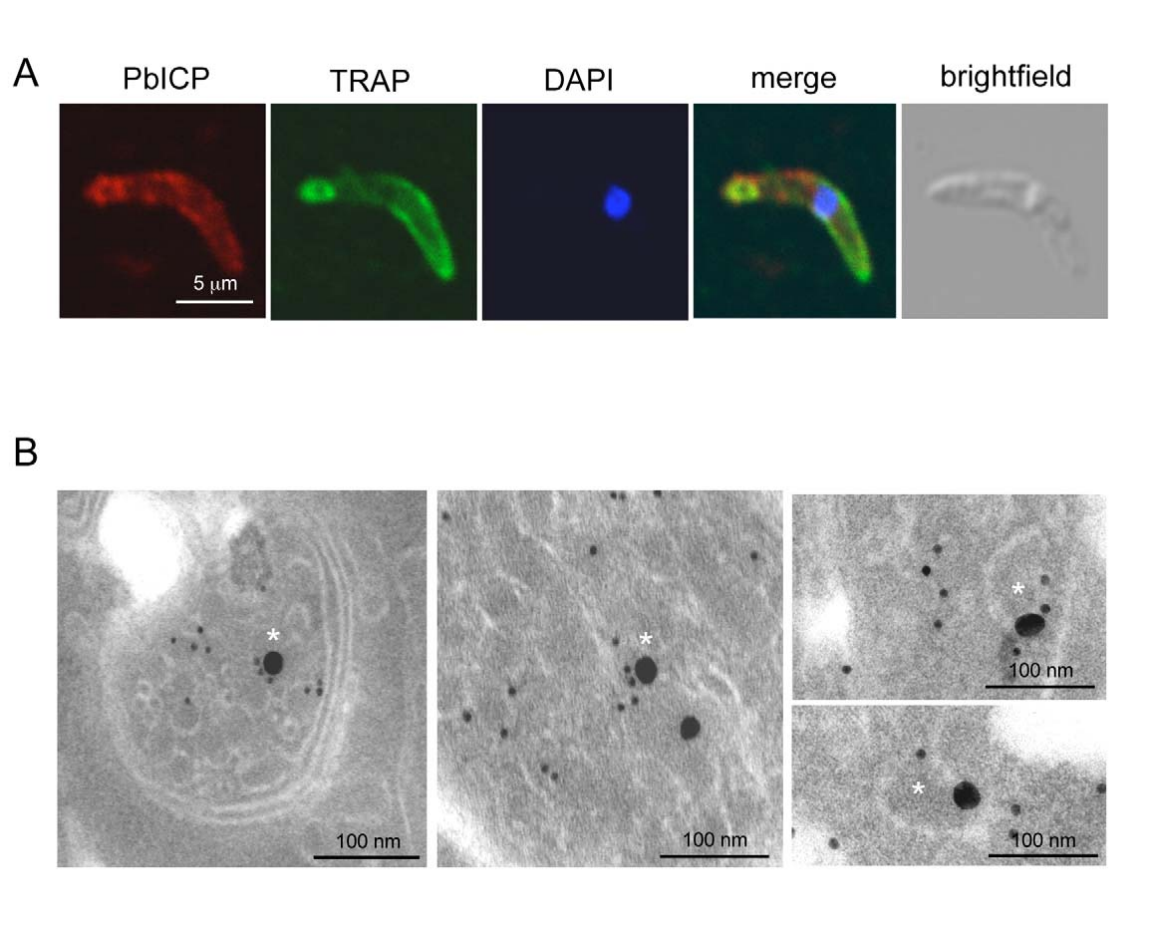
PbICP partially colocalizes with TRAP at the apical pole and in vesicles of sporozoites. (**A**) Confocal IFA of an extracellular *P. berghei* sporozoite in the presence of HepG2 cells. HepG2 cells were co-cultivated for 1 h with sporozoites, fixed and stained with an anti-TRAP antiserum (mouse, green) and a polyclonal antiserum against PbICP-C (rabbit, red). DNA was stained with DAPI (blue). (**B**) Double-stained IEM of a salivary gland sporozoite shows partial co-localization of TRAP and PbICP in vesicles (marked with asterisk). *P. berghei*-infected *A. stephensi* salivary glands were fixed 25 days after infection and prepared for IEM. Ultrathin sections were stained with PbICP-C (rabbit, 25 nm gold particles) and anti-TRAP (mouse, 10 nm gold particles). The sections were contrasted with uranyl acetate and lead citrate.

**Figure 4:**
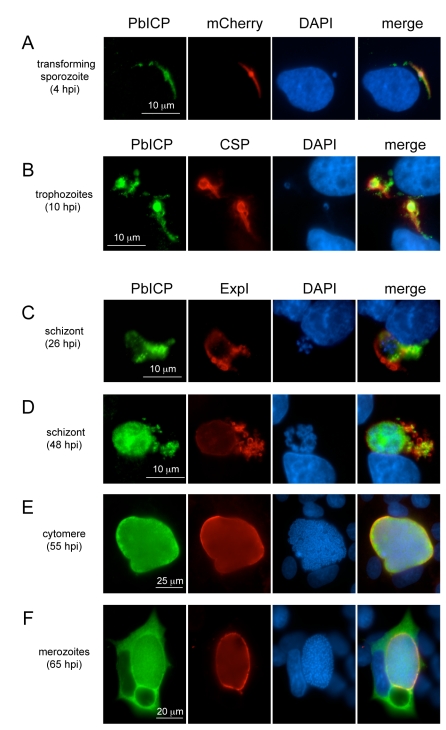
PbICP is expressed throughout the liver stage and localizes to different microenvironments. Wide-field IFA of HepG2 cells infected with *P. berghei*-expressing mCherry in the cytoplasm (**A**) or wildtype *P. berghei* (**B-F**) at different time points after infection (hpi, hours post infection). Infected cells were fixed, incubated with anti-CSP antiserum (mouse, red) (**B**) or with anti-ExpI antiserum (chicken, red) (**C-F**) and antiserum against PbICP-C (rabbit, green) (**A-F**). DNA was stained with DAPI (blue).

### Sporozoites secrete PbICP

Extracellular gliding sporozoites were fixed and stained with an antiserum directed against the C-terminal domain of PbICP, revealing that sporozoites secrete the inhibitor (Figure 2A). PbICP was detected in distinct areas of the parasite and in protein trails left behind by gliding sporozoites. These trails also contain the major sporozoite surface protein CSP (circumsporozoite protein) that is shed during gliding motility., In permeabilized sporozoites, PbICP staining showed a patchy pattern suggesting a localization in secretory vesicles, which was confirmed by IEM (Figure 2B). TRAP (Thrombospondin-related anonymous protein) is a micronemal protein and, in the presence of host hepatocytes, accumulates at the apical end of the sporozoite [59],[60]. IFA revealed a similar staining pattern for PbICP, suggesting that an exocytosis-like secretion of the inhibitor occurs as for TRAP (Figure 3A). In addition, staining of unfixed sporozoites with an anti-PbICP-C antiserum confirmed the localization at the apical pole of the sporozoites (Figure S5).

To investigate whether the PbICP-containing vesicles are micronemes, we performed double staining IEM analysis of sporozoites using TRAP as a micronemal marker protein (Figure 3B). In agreement with the confocal IFA, the IEM analysis showed a partial colocalization of TRAP and PbICP in secretory vesicles. Thus, we conclude that at least a portion of PbICP is translocated to the micronemes.

Following invasion of hepatocytes, intracellular parasites continue secreting PbICP, which was detected in the host cell cytoplasm (Figure 4A,B, Figure S6, Figure S7). To ensure we were monitoring invading and not transmigrating parasites, we fixed cells at 4 hpi (hours post infection), when sporozoites are no longer motile. At this stage, some sporozoites had already begun transformation into early trophozoites (Figure S6A). To confirm export of PbICP into the host cytoplasm, we compared the localization of PbICP with that of CSP, a molecule known to be secreted by young trophozoites. Although we did not detect co-localization of these proteins, the distribution of CSP and PbICP in the infected hepatocyte was similar for the majority of parasites, indicating PbICP secretion (Figure S6B).

### In later liver stages, PbICP is translocated into the parasitophorous vacuole and is released into the hepatocyte cytoplasm upon merozoite formation

The PVM of early schizont stages can be stained with an antiserum against the PVM-marker protein Exp1 of *P. berghei.* PbICP co-localized partly with Exp1 but was additionally clearly seen outside of the ring-shaped Exp1 staining, strongly suggesting that the inhibitor is in contact with the host cell cytoplasm (Figure 4C). In later schizont and cytomere stages, PbICP localized mainly to the PV (Figure 4D, E, Figure S4B, Figure S8, Figure S9), but was also found in the parasite cytosol. For these stages we could not detect PbICP in the host cytoplasm. However, PbICP was frequently found in close proximity to Exp1-positive structures that appear to bud off or be released from the PVM (Figure 4D, Figure S4B, Figure S8 and Figure S9).

After completion of daughter parasite development, the PVM starts to disintegrate and this clearly correlates with marked PbICP release into the hepatocyte cytoplasm (Figure 4F, Figure S10) suggesting that PbICP could have an important function in the regulation of host cell proteases and the regulated host cell death induced at this time. Upon PVM rupture and PbICP release, merozoites are liberated into the host cell cytoplasm and thus are in direct contact with PbICP, which might protect them from proteolytic damage.

### PbICP is posttranslationally processed

**Figure 5:**
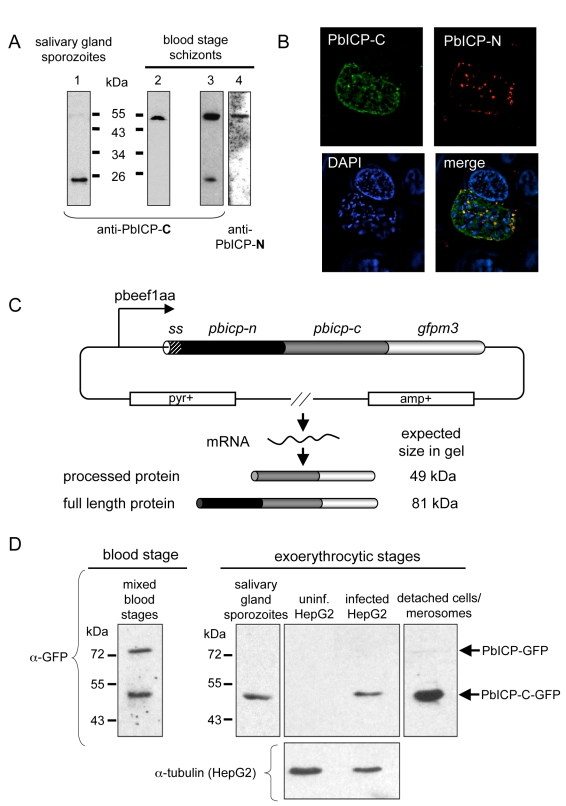
PbICP is posttranslationally processed in the blood stage and exoerythrocytic stages. (**A**) Domain-specific western blot analysis of protein extracts prepared from *P. berghei* salivary gland sporozoites and blood stage schizonts. Protein extracts of blood stage schizonts (two independent samples: one in lane 2 and a second one in lane 3+4) and sporozoites were separated by SDS-PAGE and blotted on nitrocellulose filters. Filters were then probed with antisera against the chagasin-like domain of PbICP (anti-PbICP-C, rabbit, lane 1–3) or the N-terminal domain (anti-PbICP-N, lane 4). Anti-PbICP-C antiserum detected the full-length inhibitor (55 kDa) as well as the processed form after cleavage of the N-terminal extension domain (23 kDa), whereas anti-PbICP-N antiserum detected only the full-length protein of 55 kDa. (**B**) IFA of a *P. berghei*-infected HepG2 cell 48 hpi (widefield deconvolution). Infected cells were fixed and stained with anti-PbICP-N antiserum (mouse, red) and antiserum against PbICP-C (rabbit, green). DNA was stained with DAPI (blue). The images were deconvoluted to remove out-of-focus signals. (**C**) Schematic illustration of the transfection plasmid pL0017-*pbicp-gfp* for constitutive strong expression of PbICP. The *pbicp-gfpm3* coding region (ss, signal sequence of *pbicp*) is under the control of the strong constitutive *pbeef1aa* promotor. The vector confers ampicillin resistance (amp+) in *E. coli* and pyrimethamine resistance (pyr+) in transfected *P. berghei* parasites. Following transfection, *P. berghei* parasites constitutively express the fusion protein. The expected *in gel* sizes of the GFP-tagged full-length PbICP and the GFP-tagged processed form of the inhibitor were calculated and are presented. (**D**) Anti-GFP western blot analysis of GFP-PbICP-expressing *P. berghei* blood stage extracts, sporozoite total lysates, infected HepG2 cell total lysates (60 hours after infection) and detached infected HepG2 cells/merosomes. Uninfected HepG2 cells served as a specificity control and reprobing with anti-tubulin antiserum served as a loading control for protein extracts of infected and uninfected HepG2 cells. The anti-GFP antiserum detected proteins that correspond to the size of the full-length GFP-tagged inhibitor (81 kDa) as well as the processed form after cleavage of the N-terminal extension domain (49 kDa).

Western blot analysis using anti-PbICP antisera revealed that PbICP is subject to posttranslational processing during the sporozoite stage and frequently also during the blood stage of the parasite (Figure 5A). Antisera directed against the C-terminal chagasin-like domain of PbICP not only detected a protein that corresponds to the full-length PbICP but also a 23 kDa protein that corresponds to the chagasin-like C-terminal domain of PbICP. An antiserum directed against the N-terminal extension region of PbICP only detected the full-length inhibitor, indicating that the N-terminal region is rapidly degraded after the proteolytic cleavage. While in sporozoites processing of PbICP was always detected, the situation was less clear for blood stage parasites. In protein extracts of this stage we sometimes exclusively found unprocessed PbICP (Figure 5A), similarly to what has been published for falstatin/PfICP of *P. falciparum* [40] but in other preperations processed PbICP was also detected. As both non-processing and partial processing were seen in the schizont stage, the degree of processing does not seem to relate to the developmental stage of the parasite. Since the focus of this study was on exoerythrocytic parasites, we did not follow up this interesting phenomenon in detail. Blood stage protein extracts were nevertheless used as an easily accessible source of protein for western blotting to address questions unrelated to processing. Importantly, the size of the full-length inhibitor detected by western blotting corresponds to the size of recombinant PbICP, suggesting that parasite-derived PbICP is not posttranslationally modified other than by processing (data not shown). To analyze processing of PbICP during liver stage development by western blotting, we generated a *P. berghei* strain expressing a PbICP-GFP fusion protein under the control of a strong constitutive promotor (*eef1aa*) (Figure 5C). PbICP overexpression was necessary because infection rates of HepG2 cells are in general very low (2–10%) and do not allow detection of endogenous proteins by western blotting. Using this PbICP-overexpressing transgenic strain for HepG2 cell infection, it was then possible to demonstrate PbICP processing before and after the detachment of infected hepatocytes at the end of the liver stage (Figure 5D). Overexpression of the PbICP-GFP fusion protein had no negative effects on parasite development suggesting that the presence of the fusion protein is not toxic for exoerythrocytic parasites (Figure S11A-E). Interestingly, transgenic parasites constitutively expressing PbICP-GFP showed a slightly but significantly increased level of HepG2 cell invasion compared to wildtype parasites (Figure S12). To prove PbICP processing also occurs in wildtype parasites, we employed immunofluorescence analysis using antisera against both PbICP-C and PbICP-N. We reasoned that co-localization of these domains would indicate unprocessed PbICP whereas staining exclusively with the PbICP-C antiserum would indicate PbICP processing. The result of the immunofluorescence analysis confirmed a proteolytic cleavage of endogenous PbICP during the schizont stage of the parasite (Figure 5B).

### The C-terminal domain is necessary and sufficient for the inhibitory function of PbICP

**Figure 6:**
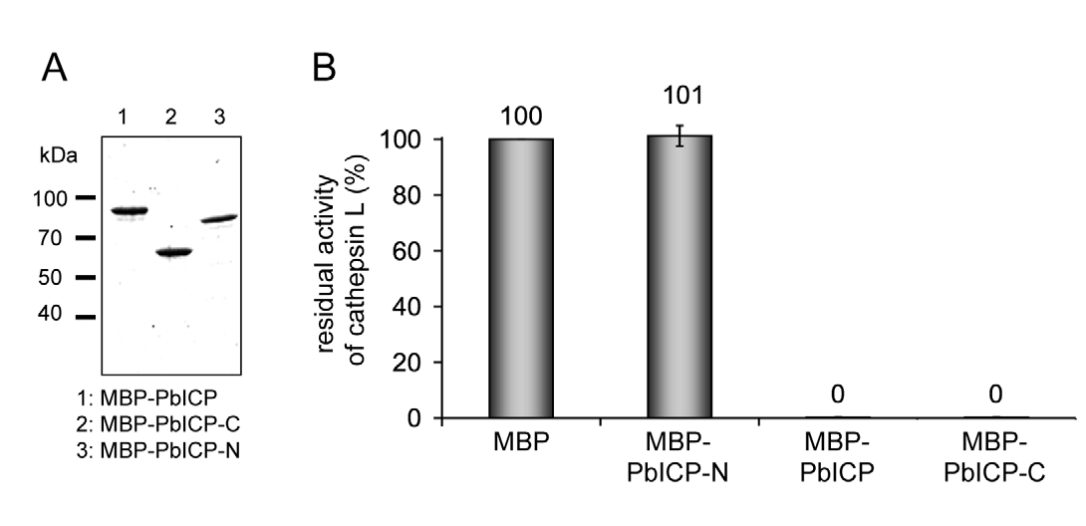
The C-terminal domain of PbICP is necessary and sufficient for the inhibitor function. (**A**) Purification of recombinant PbICP, PbICP-C and -N. Like the full-length protein, recombinant PbICP-C and -N were expressed in *E. coli* as MBP-tagged soluble proteins and purified from the bacterial total lysate by amylose-bead affinity chromatography (lane 1, 2, 3). The proteins were resolved by SDS-PAGE and stained with Coomassie Blue. (**B**) Recombinant PbICP-C inhibits the cysteine protease cathepsin L to a similar extent as full-length PbICP, whereas PbICP-N does not block cathepsin L activity. Hydrolysis of the substrate Z-FR-pNA by cathepsin L was measured in the presence of 1 µM MBP-PbICP-N, MBP-PbICP or MBP-PbICP-C or the control protein MBP.

We then investigated whether the C-terminal domain of PbICP on its own has inhibitory potential. PbICP-C, the region homologous to chagasin, was expressed as an MBP fusion protein (Figure 6A) and used in protease assays (Figure 6B, Table 2). Full-length MBP-PbICP served as a positive control, MBP without any fusion as a negative control. Additionally, PbICP-C was expressed and purified without a tag and included in the analysis. Both MBP-PbICP-C and PbICP-C blocked papain, cathepsin L and falcipain-2 activity to the same extent as full-length MBP-PbICP, whereas the tagged N-terminal domain (MBP-PbICP-N) did not show any inhibitory effect on protease activity (Figure 6B, Table 2). None of the recombinant proteins blocked the activity of cathepsin B. Since the full-length inhibitor and PbICP-C did not differ in inhibitory strength and specificity for the analyzed proteases, the N-terminal domain does not appear to have a modulatory effect on the inhibitor function at least in a non-cellular environment.

**Table 2:** The C-terminal domain but not the N-terminal domain of PbICP is a potent inhibitor of cysteine proteases except cathepsin B.

	papain	cathepsin L	falcipain-2	cathepsin B
1 µM MBP	100	100	100	100
1 µM MBP-PbICP-N	**89±1**	**101±4**	**128±2**	**93±7**
1 µM MBP-PbICP-C	**5±2**	**1±0,2**	**0±0,1**	**100±1**
100 nM MBP	100	100	100	100
100 nM PbICP-C	**12±2**	**21±10**	**18±0,1**	**105±2**

Substrate hydrolysis was measured in the presence of the control protein MBP (1 µM or 100 nM), in the presence of MBP-PbICP-C (1 µM), in the presence of MBP-PbICP-N (1 µM) or in the presence of PbICP-C (100 nM). Protease activity in the presence of 1 µM and 100 nM MBP, respectively was considered as 100% and the percentage of residual protease activity in the presence of the inhibitor was calculated.

### PbICP is involved in sporozoite invasion of hepatocytes

**Figure 7:**
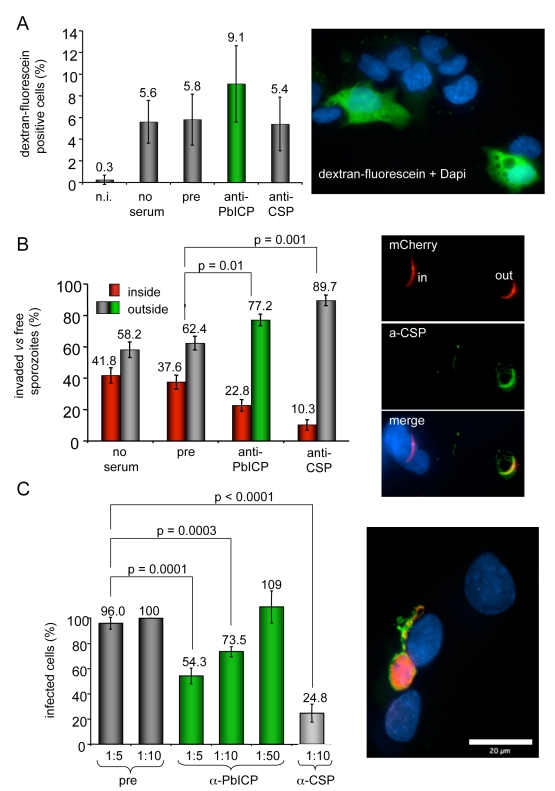
Antibody-mediated neutralization of PbICP reduces infectivity of sporozoites *in vitro* but does not inhibit cell traversal of sporozoites. (**A**) *Transmigration assay: P. berghei* sporozoites were pre-incubated with medium (no serum) preimmune serum (1∶5), anti-PbICP-C antiserum (1∶5) or anti-CSP-antiserum (1∶5) and subsequently added to HepG2 cells in the presence of 1 mM dextran-fluorescein for 1 h to stain wounded cells. Non-infected HepG2 cells (n.i.) cultivated in the presence of 1 mM dextran-fluorescein for 1 h served as a negative control for cell wounding. After this incubation period cells were washed, fixed and the DNA was stained with DAPI to allow visualization of both intact and wounded cells. The total cell number compared to dextran-fluorescein positive cells was determined using an immunofluorescence microscope and the percentage of dextran-fluorescein positive cells was calculated. Presented are the means and standard deviations of three independent experiments. The image presented shows a number of cells, whose nuclei are stained with DAPI. Transmigrated cells are additionally stained with dextran-fluorescein (green). (**B**) Invasion assay: *P. berghei* sporozoites expressing mCherry were pre-incubated with preimmune serum (1∶5), anti-PbICP-C antiserum (1∶5) or anti-CSP-antiserum (1∶5) and subsequently added to HepG2 cells. After 1 h, cells were fixed but not permeabilized and stained with an anti-CSP antiserum (inside/outside assay). All sporozoites can be detected by their mCherry expression but only extracellular sporozoites are additionally stained by the anti-CSP antiserum. DNA was stained with DAPI (blue). Free and intracellular sporozoites were counted and the percentage was calculated. Presented are the means and standard deviations of three independent experiments. Typical examples of intracellular and extracellular parasites are presented in the image next to the graph. mCherry expression can be seen in all sporozoites but additional CSP staining (green) is visible only in extracellualr parasites. (**C**) Parasite development following PbICP neutralization of sporozoites: HepG2 cell infection was performed as described in (B). Serum dilutions (1∶5 to 1∶50) used to pre-incubate sporozoites are indicated. Infected cells were fixed 30 hpi and stained with anti-Exp1 antiserum (chicken, green) and anti-PbICP-C antiserum (rabbit, red) (image shows a typical example of a infected cell 30 hpi stained as described). Infected cells in the individual wells were counted and the percentage of infected cells after treatment was calculated from three independent experiments. The number of infected cells in wells infected with sporozoites that had been exposed to a 1∶10 dilution of the preimmune serum, was considered as 100%. As in the CSP-control, pre-incubation with anti-PbICP-antiserum significantly reduced the infectivity of sporozoites.

Since PbICP is secreted by sporozoites, we tested whether the protein plays a role during sporozoite transmigration through cells and invasion of HepG2 cells *in vitro* (Figure 7). Sporozoites were pre-incubated in anti-PbICP antiserum and then used for the different assays. Interestingly, the number of transmigrated cells was higher for sporozoites pre-incubated with anti-PbICP antiserum compared to sporozoites pre-incubated with anti-CSP antiserum or preimmune serum (Figure 7A). However, since this effect was not significant, we did not follow up this observation. The most important conclusion from the transmigration assay was that neutralization of PbICP does not interfere with the traversal capability of sporozoites. Following transmigration, sporozoites finally invade cells and reside in a PV. To investigate the effect of PbICP blockage on the invasion process, we combined the invasion assay with an inside/outside assay that allowed us to distinguish between extracellular and intracelluar parasites [5] (Figure 7B). Preincubation of sporozoites with antiserum against PbICP-C significantly reduced the level of HepG2 cell infection by *P. berghei* sporozoites by about 40% (from 37.6% to 22.8% in Figure 7B). The inhibitory effect on sporozoite invasion by blocking PbICP activity fits well with the observation that transgenic *P. berghei* parasites over-expressing a GFP-PbICP fusion protein exhibited an improved invasion rate (Figure S12).

To exclude an effect of PbICP neutralization during invasion on parasite development, we additionally analyzed infected cells 30 hpi. The number of parasites that developed to the schizont stage (see image in Figure 7C) was significantly reduced by 46% or 26% when sporozoites were pre-treated with 1∶5 or 1∶10 dilutions, respectively, of the anti-PbICP antiserum in comparison to the pre-immune control serum (Figure 7C). The reduction of developing parasites by 46% after pretreatment of sporozoites with 1∶5 dilution of the serum reflects the result of the inside/outside assay, where the number of invaded sporozoites was reduced by almost the same extent (40%). In conclusion, the blocking capacity of the antiserum is restricted to sporozoite neutralization and does not affect later parasite development. As expected, preincubation of sporozoites in 1∶10 diluted anti-CSP antiserum resulted in a strong (75%) reduction of the number of infected HepG2 cells 30 hpi.

### PbICP blocks host cell death

**Figure 8:**
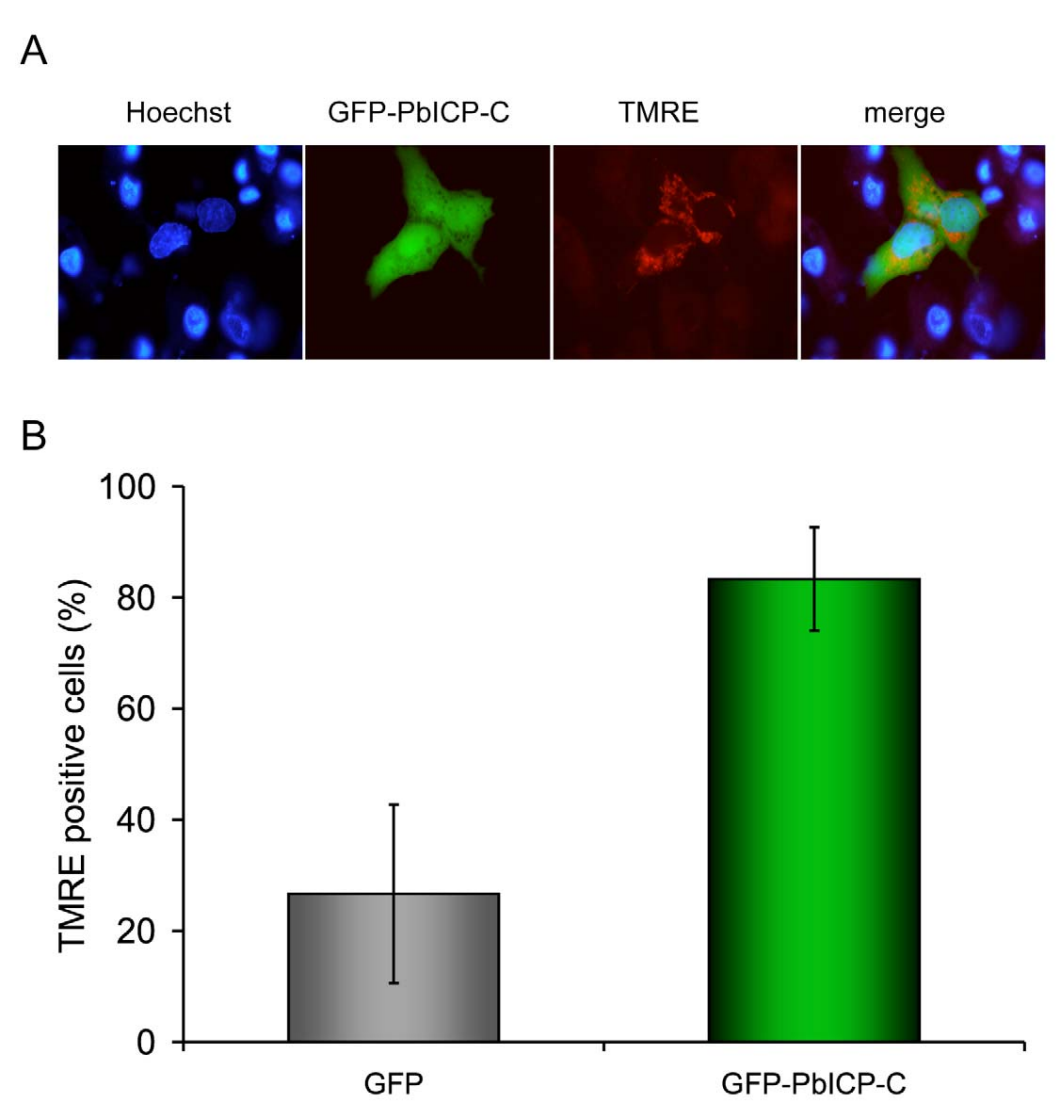
PbICP-C expression protects HepG2 cells against host cell death. (**A**) HepG2 cells were transiently transfected with plasmids leading to either cytosolic expression of GFP-tagged PbICP-C or GFP as a control. The transfection efficiency of HepG2 cells with the control plasmid and the GFP-PbICP-C plasmid (absolute numbers of GFP-positive cells/cover) were similar. Host cell death was induced by tBHP treatment for 4 h and analyzed by live imaging using TMRE staining of intact mitochondria (red). DNA condensation was visualized by Hoechst 33258 staining (blue). Dying cells exhibited condensed chromatin in the nucleus and a loss of the mitochondrial membrane potential. (**B**) Fluorescent cells were counted and the percentage of dead and viable cells was calculated. Presented are the means and standard deviations of three independent experiments. Cells expressing GFP-PbICP-C showed a significantly better survival following tBHP treatment in comparison to GFP-expressing cells.

**Figure 9:**
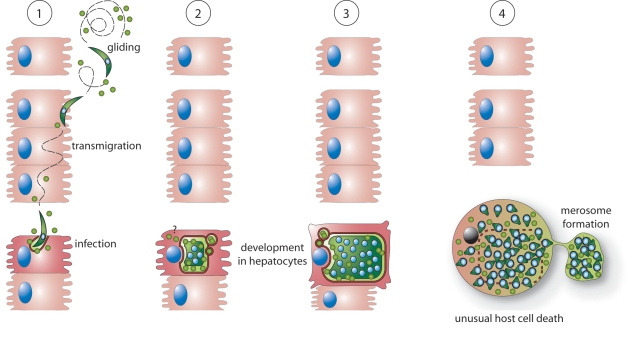
Possible roles of PbICP during the exoerythrocytic development of *P. berghei in vitro*. **1:** PbICP (green circles) is secreted by free gliding sporozoites and supports invasion of HepG2 cells. Intracellular sporozoites and trophozoites continue to express and partially secrete PbICP. Thus PbICP can potentially control parasite-derived cysteine proteases as well as host cell cysteine proteases. **2+3:** During schizogony, PbICP is predominately located in the PV and the parasite cytosol, suggesting that during this developmental phase, parasite cysteine proteases are the main target of the inhibitor. However, it cannot be excluded that small portions of the inhibitor, which are beyond the detection level for IFA, are still exported into the host cell cytoplasm. **4:** At the end of the liver stage, upon disruption of the PVM, a substantial amount of PbICP is passively released into the host cell cytoplasm. At this stage, the main function of PbICP might be inhibition of host cell cysteine proteases to allow a slow and ordered host cell death and merosome formation.

Since PbICP is released into the host cell cytoplasm both by sporozoites and at the end of the liver stage, the question is raised whether host cell cysteine proteases are target molecules of the parasite inhibitor. Cysteine proteases such as caspases, calpains and cathepsins are often key enzymes in programmed cell death execution. Interestingly, when host hepatocytes undergo their unusual cell death upon merozoite release from the PVM, caspases are not activated and other classical signs of programmed host cell death are also absent, including DNA fragmentation and phosphatidylserine switching to the outer plasma membrane leaflet [16]. We hypothesized that PbICP may modulate activation of programmed host cell death by inhibiting host cell cysteine proteases. HepG2 cells were transfected with a plasmid coding for N-terminally GFP-tagged PbICP-C or with a control vector and subsequently cell death was induced by treatment with tert-butyl hydroperoxide (tBHP). Viability of cells was analyzed by staining with TMRE, a dye which only labels mitochondria with intact membrane potentials and thus indicates viable cells. Additionally, cells were stained with the DNA dye Hoechst 33258 to visualize chromatin condensation of dying cells (Figure 8A). In contrast to the control cells expressing GFP, of which only 26% were alive, after 7 h of tBHP treatment, 80% of the GFP-PbICP-C- expressing HepG2 cells were still viable (Figure 8B). Similar results were achieved when infected cells were treated with the apoptosis-inducing agent camptothecin (CAM) for 48 h (Figure S14). From these experiments we conclude that PbICP indeed has the capacity to suppress or interfere with the cell death machinery of hepatocytes by blocking host cell cysteine proteases involved in cell death execution. The possible function of PbICP during the entire exoerythrocytic development *in vitro* is summarized in Figure 9.

## Discussion

Parasite and host cell cysteine proteases play an important role during the entire life cycle of the *Plasmodium* parasite. Cysteine protease activity is essential for sporozoite egress from oocysts in the mosquito [19], sporozoite invasion of hepatocytes in the vertebrate host [20],[21], liver stage development and liberation of exo-erythrocytic merozoites from hepatocytes [16] as well as for invasion, nutrition and egress in the blood stage [18]. All these protease activities must be strictly regulated to prevent undesired and harmful proteolytic activity. In addition to controlling its own proteases, the malaria parasite is exposed to proteases of the host, such as those from inflammatory cells that are secreted to eliminate pathogens and perhaps also intra-hepatocyte proteases encountered by the parasite during liver stage development.

In this study, we report the identification of the potent cysteine protease inhibitor PbICP, the falstatin/PfICP homolog of the rodent malaria parasite *P. berghei*. Like falstatin/PfICP [40], PbICP is an unusual member of the chagasin inhibitor family with obvious structural peculiarities due to (1) the long N-terminal extension domain, (2) sequence insertions in the chagasin-like C-terminal domain and (3) amino acid exchanges in conserved motifs. The inhibitor is expressed in all analyzed stages of *P. berghei* (blood stage, sporozoites, liver stages) and according to its different localizations it can potentially control parasite as well as host cell-derived proteases.

We provide several lines of evidence that PbICP is secreted by *P. berghei* sporozoites to support host cell invasion. First, the inhibitor was detected in typical CSP-positive sporozoite trails [61] by IFA of fixed parasites using a specific anti-PbICP antiserum. Second, IEM analysis revealed the presence of PbICP in vesicles within sporozoites. Third, some of the PbICP-positive vesicles appear to be secretory micronemes since they were found by IEM analysis to be positive for both PbICP and the micronemal marker protein TRAP [62],[63],[64],[65]. Fourth, IFA of live sporozoites revealed PbICP to be in a characteristic protein cap formation at the apical pole, as has been seen for the TRAP protein after regulated exocytosis [59],[60]. Finally, pre-incubation of viable sporozoites in anti-PbICP immune serum significantly reduced the number of sporozoites invading HepG2 cells.

PbICP does not contain an obvious micronemal targeting motif but since these motifs are rather heterogenous (as in TRAP and EBL proteins [62],[66]), empirical studies will be necessary to determine the motif that targets PbICP to the micronemes of the sporozoite. As a soluble protein, PbICP may even lack such a motif but might be sorted by formation of complexes with escorter proteins, as is known for soluble micronemal MIC proteins of *Toxoplasma* [67].

Our results are supported by a previous study on the *Plasmodium gallinaceum* ICP [68], which was termed PgSES for *P. gallineceum* sporozoite and erythrocyte stage protein. In this study, PgSES/PgICP was not identified as a cysteine protease inhibitor but was characterized as a secreted sporozoite protein with a characteristic localization pattern. Similar to what we describe for *P. berghei* sporozoites, LaCrue *et al.* found a patchy intracellular distribution of PgSES/PgICP in the sporozoite and an extracellular staining pattern distinct from CSP, mainly on salivary gland sporozoites [68].

The target protease of sporozoite-secreted PbICP remains to be identified, but interestingly it was reported that a cysteine protease-dependent CSP cleavage is essential for the invasion process [20],[21]. The responsible protease has not yet been identified but is of parasite origin and becomes activated upon interaction of the parasite with highly sulfated heparan sulfate proteoglycans (HSPGs) on hepatocytes. Since PbICP is capable of regulating cysteine proteases, and since both molecules act outside of the sporozoite, it is possible that PbICP controls this parasite protease in a timely and well-orchestrated manner to avoid premature CSP proteolysis.

When sporozoites were pre-incubated with anti-PbICP antiserum, we not only observed a reduced invasion rate but also a tendency of enhanced transmigration. It has been suggested earlier that sporozoites which are blocked from invasion persist in the migratory state [21] and it might well be that we provoked a similar effect by neutralizing the protease inhibitor.

In agreement with our findings that PbICP contributes to hepatocyte infection by *P. berghei* sporozoites, many of the so far characterized chagasin-like inhibitors of other intracellular protozoan parasites also play an important role during host cell invasion processes. Falstatin/PfICP is released together with *P. falciparum* merozoites upon rupture of infected red blood cells. Treatment with antibodies directed against falstatin/PfICP decreased the subsequent invasion of erythrocytes in a dose-dependent manner, suggesting a role in limiting unwanted proteolysis during erythrocyte invasion [40]. The ICPs of *Trypanosoma* are predicted to regulate endogenous proteases of the parasite [45],[46]. Amongst other effects on differentiation and protein turnover, overexpression of chagasin in *T. cruzi* resulted in a reduced infection rate *in vitro,* while *T. brucei* ICP null mutants reached higher parasitemia levels in mice [45],[46]. Endogenous *L. mexicana* ICP had no impact on the infectivity of the parasites *in vitro.* However, when LmICP null mutants and overexpressors were analysed *in vivo* in the mouse model system, both showed a reduced virulence and infectivity [38]. It is suggested therefore, that LmICP controls host-derived rather than parasite-derived proteases.

Upon sporozoite invasion of hepatocytes and transformation into trophozoites, PbICP is still secreted by the parasite, entering the PV and apparently reaching the host cell cytoplasm. Since a PEXEL export motif is absent in PbICP, it is so far unknown how the inhibitor crosses the PVM. It has been shown previously that *P. berghei* infection protects host cells from apoptosis [14],[15] and PbICP translocated to the host cell cytoplasm might be involved in the inhibition of caspases, which are cysteine proteases involved in programmed cell death execution. Indeed, we provide the first evidence that PbICP expression in the host cell cytoplasm is sufficient to inhibit parasite-independent host cell death. Interestingly, Pandey *et al.* have shown that falstatin/PfICP efficiently blocks proteolytic activity of caspases in nano- to micromolar concentrations [40]. It remains to be shown how PbICP could act on enzymes as different as cathepsin-L-like proteases and caspases. For C1 family members such as cathepsins, it is known that ICPs bind to the active site cleft between the R and the L domain, but caspases do not contain such a cleft [69]. A possible explanation is that *Plasmodium* ICPs act similarly to members of the serpin family due to the sequence insertions, which lead to elongated loops. Serpins inhibit serine and cysteine proteases by using a flexible loop (reactive site loop) as a bait to trap structurally different proteases [70]. If *Plasmodium* ICPs indeed function similarly to serpins, this would explain why falstatin/PfICP is an efficient inhibitor of structurally different proteases.

At the end of the liver stage, the situation changes completely and an ordered parasite-mediated cell death is induced. This form of cell death clearly differs from apoptosis since caspases are not involved, there is no switch in phosphatidylserine residues to the outer leaflet of the host cell membrane and the dying cell does not shrink or form apoptotic bodies but rather expands in size [16]. Since this parasite-induced cell death can be inhibited by treatment with the cysteine protease inhibitor E64, cysteine proteases appear to be key players in this process. We found considerable amounts of PbICP in the host cell upon PVM breakdown. Thus, it can be predicted that the cysteine proteases responsible for cell death induction belong to the cathepsin-B type that is not targeted by PbICP. Potential candidates are SERA proteases, a family of parasite-derived putative cysteine proteases. They are released into the host cell cytoplasm at the same time as PbICP. Unfortunately, experimental proof of the hypothesis that PbICP does not inhibit SERA protease activity is not possible because SERA proteases cannot currently be recombinantly produced in their enzymatically active state. Why would the parasite not simply allow the host cell to undergo apoptosis but instead use its own proteases to induce host cell death? The answer might be that activation of apoptosis-related proteases such as caspases and calpain-1 induces a rapid destruction of the cytoskeleton and a breakdown of the cell into apoptotic bodies or rupture of the host cell membrane. A rapid destruction of the host cell would result in a premature release of merozoites before they have been transported inside merosomes safely into the blood vessels. Merosome formation and merozoite transport can last for several hours and thus the parasite needs to maintain control of host cell proteases. On the other hand, the parasite requires cysteine proteases that induce an ordered but slow host cell death and allow the formation of merosomes.

To further analyze the function of PbICP translocated into the host cell cytoplasm, it will be necessary to identify its target protease. We suggest that PbICP acts on both parasite and host cell proteases. An effect on host cell proteases has already been shown for a number of ICPs of other pathogens. A good example is the prokaryote *P. aeruginosa,* which expresses a functional chagasin homolog but no potential target proteases of the C1 protease family [37], suggesting that chagasin-like inhibitors in general may have evolved to inhibit foreign proteases.

All so-far characterized chagasin-like inhibitors have shown a significantly lower affinity to cathepsin B than to cathepsin L-like C1 cysteine proteases [37],[58]. For chagasin, it was shown that this decreased cathepsin B affinity is due to the occluding loop that is in close proximity to the active site of the protease [58] [71]. In contrast to the so-far characterized chagasin members of other organisms, the *Plasmodium* ICPs show a complete loss of cathepsin B inhibition [40]. *Plasmodium* ICPs contain sequence insertions in the chagasin-like C-terminal domain, which might, in principle, interfere with cathepsin B binding. A recent publication reports that *T. gondii*-derived toxostatins, two other members of the chagasin family, contain similar extensions as found in the chagasin-like domain of PbICP. Remarkably, toxostatin-1 was already shown to retain the capability of inhibiting cathepsin B [43]. Together with our own data, it can be concluded that the lack of cathepsin B inhibition by *Plasmodium* ICPs is not caused by an interference of the extension loops with the occluding loop of the protease but must be due to other structural motifs.

To characterize the function of PbICP, we generated transgenic *P. berghei* parasites constitutively over-expressing a GFP-tagged version of the inhibitor. Although these parasites were very helpful for analyzing PbICP localization and processing during the liver stage, the genetic manipulation did not provoke a pronounced phenotype apart from a slightly better invasion rate. The best genetic manipulation to analyze the biological function of PbICP would be to knock out the *pbicp* gene. This approach has been tried several times without success, strongly suggesting that PbICP expression is essential during the blood stage because transfection and the selection of transgenic parasites is performed at this stage.

In conclusion, we identified a potent cysteine protease inhibitor of *P. berghei* that seems to play different roles during the life cycle of the malaria parasite. In the exo-erythrocytic stage, PbICP is important for the invasion of sporozoites and is able to protect the host cell from apoptosis, which is essential for the completion of liver stage development.

## Supporting Information

Figure S1 Multiple sequence alignment of ICPs. Multiple sequence alignment of PbICP and the ICPs of *P. yoelii* (PyICP) and *P. falciparum* (falstatin/PfICP) in comparison with the ICPs of *T. gondii* (toxostatins), *T. cruzi* (chagasin), *T. brucei, L. mexicana* and the two ICPs of *E. histolytica*. Conserved amino acid residues of the chagasin inhibitor family are highlighted in grey, the wedge forming loops that bind the active-site cleft of proteases (L2, L4, L6) are highlighted in yellow. The amino acid residues of chagasin highlighted in purple form the β-sheet strands. The N-terminal residues of PbICP highlighted in pink represent the classic signal sequence.
(0.56 MB PDF)

Figure S2 Multiple sequence alignment of the ICP chagasin domains. (A) Multiple sequence alignment of the C-terminal chagasin-like domain of PbICP, PyICP and falstatin/PfICP in comparison with the ICPs of *T. gondii* (toxostatins), *T. cruzi* (chagasin), *T. brucei, L. mexicana* and the two ICPs of *E. histolytica*. Conserved residues of the chagasin inhibitor family are highlighted in grey, the wedge forming loops that bind the active-site cleft of proteases (L2, L4, L6) are highlighted in yellow. At the top of the alignment the β-strands of chagasin are displayed in purple. The amino acid sequences of chagasin that form β-strands are additionally indicated in purple. (B) Known β-strands of chagasin and predicted β-strands of PbICP are depicted by arrows. In contrast to chagasin (purple), the inhibitor domain of PbICP (green) is predicted to have two additional β-strands (β5′ and β5′′) and elongated loop-structures L3 and L4 (red, dashed line) as well as a N-terminal extension region with a classic N-terminal signal sequence (grey). The wedge-forming loops that bind into the active site cleft of the proteases (L2, L4, L6) are highlighted in yellow. Like the toxostatins of *T. gondii*, but in contrast to the non-apicomplexan chagasin-like inhibitors of other protozoa and bacteria, the *Plasmodium* ICPs do not contain the NPTTG motif in L2 (variable motifs of *P. berghei* and *P. falciparum* are shown).
(0.31 MB PDF)

Figure S3 Schematic overview of PbICP constructs and epitopes used for the generation of antisera and examples of specificity controls. (A) To generate a specific antisera different regions of PbICP were used to immunize mice and rabbits. A mouse anti-PbICP antiserum was generated using MBP-tagged full-length PbICP. Recombinant His-PbICP-C^GDEK^ was used for immunization to produce PbICP-C domain-specific antisera. Anti-PbICP-N domain-specific antisera were obtained from mice using recombinant MBP-PbICP-N^SFNH^ for immunization and from rabbits using the peptide EDIEDNQKYPTTSYN. Panels (B and C) show a specificity control of the anti-PbICP-C antiserum (rabbit) in IEM.
(7.94 MB PDF)

Figure S4 Specificity test of anti-PbICP-antiserum directed against His-PbICP-C^GDEK^. Confocal images of HepG2 cells infected with *P. berghei* wildtype parasites 55 hpi. Preimmune control (A) and anti-PbICP-C (B). Infected cells were fixed, incubated with a chicken anti-ExpI antiserum (secondary antibody: anti-chicken Alexa 594) and a rabbit antiserum against PbICP-C (secondary antibody: anti-rabbit Cy2) (B) or preimmune serum (secondary antibody: anti-rabbit Cy2) (A). DNA was stained with DAPI (blue).
(1.67 MB PDF)

Figure S5 Staining of unfixed sporozoites shows PbICP localization at the apical pole of the sporozoite. Salivary gland sporozoites expressing mCherry were incubated on ice with rabbit anti-PbICP-C antiserum (A), rabbit anti-CSP antiserum (B) or rabbit preimmune serum (C), washed, subsequently stained with Cy2-conjugated secondary anti-rabbit antibody (green) and Hoechst 33258 (blue), again washed and immediately analyzed by fluorescence microscopy.
(0.48 MB PDF)

Figure S6 PbICP is secreted by intracellular trophozoites (confocal IFA). (A) IFA of a GFP-expressing HepG2 cell infected with *P. berghei*. Infected cells were fixed 4 hpi, incubated with polyclonal antisera against PbICP-C (rabbit) and against GFP (mouse) and subsequently stained with fluorescently labeled secondary antibodies (anti-rabbit, red and anti-mouse, green). DNA was stained with DAPI (blue). Partial co-localization of PbICP and GFP confirmed secretion of the inhibitor in the host cell cytoplasm. (B) Quantitative analysis of PbICP secretion. IFA of a HepG2 cell infected with *P. berghei*. Infected cells were fixed 4 hpi, incubated with polyclonal antiserum against PbICP-C (rabbit) and against CSP (mouse) and subsequently stained with fluorescently labeled secondary antibody (anti-rabbit conjugated with Cy2, green and anti-mouse conjugated with Alexa594, red). DNA was stained with DAPI (blue). Parasites associated with HepG2 cells and found in the same focal plane as the host cell nucleus were considered intracellular (see typical confocal images in inserts). Intracellular parasites were counted and the absolute numbers of parasites secreting either PbICP and CSP or CSP alone are shown in the graph.
(1.76 MB PDF)

Figure S7 PbICP is secreted by intracellular sporozoites (widefield IFA). IFA of HepG2 cells infected with *P. berghei* (cytosolic mCherry expression, red) 2 hours after infection. Infected cells were fixed, incubated with polyclonal antiserum against PbICP-C (rabbit) and subsequently stained with fluorescently labeled secondary antibody (anti-rabbit conjugated with Cy2, green). DNA was stained with DAPI (blue).
(1.61 MB PDF)

Figure S8 PbICP partially co-localizes with the PVM marker ExpI at the schizont stage (confocal IFA). Confocal IFA of *P. berghei*-infected HepG2 cells. Cells were fixed 30 hpi and stained with polyclonal antisera against PbICP-C (rabbit, red) and ExpI (chicken, green). DNA was stained with DAPI (blue).
(0.35 MB PDF)

Figure S9 PbICP localizes to vesicular structures in the PV of liver stage schizonts. IFA of HepG2 cells infected with *P. berghei* at 48 hpi. Infected cells were fixed and stained with anti-ExpI antiserum (chicken, red) and polyclonal antiserum against PbICP-C (rabbit, green). DNA was stained with DAPI (blue). Representative images are presented in A-D.
(2.00 MB PDF)

Figure S10 PbICP is released into the host cell cytoplasm at the end of the liver stage. IFA of HepG2 cells infected with *P. berghei* at the end of the liver stage (63 hpi) prior to and after visible destruction of the PVM. Infected cells were fixed, stained with DAPI (A) and with anti-ExpI antiserum (chicken, red) and polyclonal antiserum against PbICP-C (mouse, green) (B). Different phenotypes are presented as a cartoon (C). Late schizont/merozoite stages were counted and the percentage of each different phenotype was calculated. Presented on top of the images are the means and standard deviations of three independent experiments (frequency of phenotypes). Main phenotypes are parasites with intact PVM and PbICP restricted to the parasite and the PV, and parasites with disrupted PVM visible by Exp1 staining and PbICP release into host cell cytoplasm. hc: host cell.
(3.02 MB PDF)

Figure S11 Characterization of the PbICP-GFP-expressing liver stage parasites. (A-E) Live imaging of PbICP-GFP-expressing liver stage parasites confirmed the PbICP localization determined by the antisera-based analysis. HepG2 cells were incubated with PbICP-GFP-expressing *P. berghei* parasites and analyzed at different time points after infection. The sporozoite shown in panel (A) revealed an apical accumulation of the GFP fluorescence (marked with an asterisk). Early liver stage parasites (B) released GFP-positive structures (marked with arrows). In schizont stages (C, D), GFP fluorescence was found in the PV and the parasite cytosol. At the end of the liver stage, after detachment of the infected HepG2 cell (E), GFP fluorescence was found in the host cell cytoplasm and in the merozoites.
(3.78 MB PDF)

Figure S12 PbICP-GFP-expressing *P. berghei* show slightly enhanced infection efficiency. HepG2 cells were infected with transgenic PbICP-GFP sporozoites or GFPcon sporozoites as a control, incubated for 1 h, subsequently fixed without permeabilization and stained with an anti-CSP antiserum (inside/outside assay). Extracellular but not intracellular sporozoites were labeled by the anti-CSP antiserum. Intracellular sporozoites are only positive for GFP expression. Sporozoites were counted and the percentages of free and intracellular sporozoites were calculated. Presented are the means and standard deviations of three independent experiments.
(0.09 MB PDF)

Figure S13 PbICP-GFP expressing parasites do not differ in their intrahepatic development from mCherry-expressing parasites. HepG2 cells were infected with transgenic PbICP-GFP sporozoites or mCherry-expressing sporozoites. Parasite size was determined over the course of development in HepG2 cells using the density slice module of the OpenLab 5.03 software. Size was measured at 24, 48 and 63 hpi by live imaging. Since mCherry expression is restricted to the parasite cytosol but PbICP-GFP is also translocated into the PV, PbICP-GFP-expressing parasites appear slightly bigger (upper panel). To analyze this observation in more detail, infected cells were fixed and stained with an anti-Exp1 antiserum, which labels the PVM of both parasite strains (lower panel). In contrast to the live imaging, this experiment revealed that mCherry parasites are slightly bigger confirming that PbICP-GFP is secreted into the PV.
(0.38 MB PDF)

Figure S14 PbICP-C expression protects HepG2 cells against host cell death (camptothecin treatment). (A) HepG2 cells were transiently transfected with a plasmid leading to cytosolic expression of GFP-tagged PbICP-C (upper panel) or with a GFP control plasmid (lower panel). Subsequently host cell death was induced by camptothecin treatment for 24 h and analyzed by live imaging of intact mitochondria by TMRE (red) and DNA by Hoechst (blue). Dying cells exhibited condensed chromatin in the nucleus and a loss of mitochondrial membrane potential. (B) Fluorescent cells were counted and the percentages of dead and viable cells were calculated. Cells expressing GFP-PbICP-C showed significantly better survival upon camptothecin-induced cell death in comparison to GFP-expressing cells.
(1.28 MB PDF)
