# Supplementary material for: Exoerythrocytic Plasmodium Parasites Secrete a Cysteine Protease Inhibitor Involved in Sporozoite Invasion and Capable of Blocking Cell Death of Host Hepatocytes
Source: PLoS Pathog. 2010 Mar 26;6(3):e1000825. doi: 10.1371/journal.ppat.1000825 (PMC2845656; doi:10.1371/journal.ppat.1000825)
Supplement: Figure S1 — Multiple sequence alignment of ICPs. Multiple sequence alignment of PbICP and the ICPs of P. yoelii (PyICP) and P. falciparum (falstatin/PfICP) in comparison with the ICPs of T. gondii (toxostatins), T. cruzi (chagasin), T. brucei, L. mexicana and the two ICPs of E. histolytica. Conserved amino acid residues of the chagasin inhibitor family are highlighted in grey, the wedge forming loops that bind the active-site cleft of proteases (L2, L4, L6) are highlighted in yellow. The amino acid residues of chagasin highlighted in purple form the β-sheet strands. The N-terminal residues of PbICP highlighted in pink represent the classic signal sequence. (0.56 MB PDF) [file ppat.1000825.s001.pdf]

```
PbICP          MKSITFFVFNICSILALLSHCEDNDIYSFDIVNETNWLKIAKNIFKGKSPSNFTIIPFNN 60
PyICP          MKSITFFVFNICSILALLSHCEDRDLYSFDIVNETSWLKIAKKIFKGKSPSNFTIIPFNN 60
Falstatin      MNLLVFFCFFLLSCIVHLSRCSDNNSYSFEIVNRSTWLNIAERIFKGNAPFNFTIIPYNY 60
Toxostatin-1   MLAPTFFFP------VPLFLCDP--CFAFANKRKACGFFSCDFLLRSPEMFQLRLSVF-- 50
Toxostatin-2   MQTLKLSRALMPLTVVAFFLLSFSVASCRQGTSPRAGTAGDGETCTETPVISFLPVSGPE 60

PbICP          TGSSNDNESNKEESVLLIRKKIKS---------------------NKNHDSSII------  93
PyICP          TGSSDNNEGDKEESVLLIRKKIKS---------------------NTKHGSNII------  93
Falstatin      VNNSTE-ENNNKDSVLLISKNLKNSSNPVDENNHIIDSTKKNTSNNNNNNSNIVGIYESQ 119
Toxostatin-1   --------------LLLVLSATTS------------------------------------  60
Toxostatin-2   HPAGGS---QRDDA----------------------------------------------  71

PbICP          -SGDTVNGDIS---DLNYTASNFSDN----------SEDIEDNQKYPTTSYNS------- 132
PyICP          -SDDSVNDDISNL-SLNSTASNFSDN----------NEEIEDNQKYPTTSYNS------- 134
Falstatin      VHEEKIKEDNTRQDNINKKENEIINNNHQIPVSNIFSENIDNNKNYIESNYKSTYNNNPE 179
Toxostatin-1   -------------------------------------------LCPSACVHG-------  69
Toxostatin-2   ------------------------------------------------------------

PbICP          ------FNHLNSNIAFNEESEYI-----EINSESDLENKIKDINIKSNLEENNTMNESG- 180
PyICP          ------FNDPNSNISFNEESEFS-----EIDSESNLENNIKDINIKNNLEENNTMNEIDN 183
Falstatin      LIHSTDFIGSNNNHTFNFLSRYNNSVLNNMQGNTKVPGNVPELKARIFSEEENTEVESA- 238
Toxostatin-1   ------------------------------AESPTMEEKVKTVTFTDPP-----------  88
Toxostatin-2   ------------------------------------------------------------

PbICP          KVDSKYELTGDEKCGKSLKLGNISNQTNQETITQSLSVGEILCIDLEGNAGTG-YLWVLLG 240
PyICP          KVDSKYELTGDEKCGNSLKLGNISNQTSQETINQSLSVGETFCIDFEANAGTG-YIWALLG 243
Falstatin      ENNHTNSLNPNESCDQIIKLGDIINSVNEKIISINSTVNNVLCINLDSVNGNG-FVWTLLG 298
Toxostatin-1   ------------------------HDKVYTTTVNVSSIPAHLKLLIESSAGSGGYAFMAHD 125
Toxostatin-2   ---------------ALEAGARTIPKIHFAHGRARAAQEKPLKVEFPATLGTG-YTLVVLD 116
LmICP          --------------MIAPLSVK-DND-----KWVDTHVGKTTEIHLKGNPTTG-YMWTRVG  40
TbICP          --------------MSHNLFTEEDNN-----KTIRMVIGETFTIELESNPTTG-YTWLRSG  41
Chagasin       -------------- MSHKVTKAHNG-----ATLTVAVGELVEIQLPSNPTTG-FAWYFEG  40
EhICP-1        -----------------MSLTEDNNN-----TTITIAKGENKEIILHGNPTTG-YSWVVDS  38
EhICP-2        -MKQFIFFALLCTSTYAAIHILTEKE---DHATLHISFNDLIKIQLRTNPSTG-YAWNIEY  56

PbICP          IHKDEPIINPENFPTKLTKKSFFSEEISVTQPKKY-KIDEHDSSKNVNREIESPEQKESD 299
PyICP          VHKNEPIINPENFPTKLTKKPYFSEEISVTQPKRY-KIDEHDSSKNVDKENESQDQKESD 302
Falstatin      VHKKKPLIDPSNFPTKRVTQSYVSPDISVTNPVPIPKNSNTNKDDSINNKDGSQNNTTTN 358
Toxostatin-1   VLKG------INRQPSLELEPLAEDEMRERLQRSVSKHGVTVGKPAIEHMKN-------- 171
Toxostatin-2   VYRGLDFPLSFRDEIRQKLGGKRGRDAPLTPASEQ-KEEKQEEKAKTEGETSGEDGDIED 175
LmICP          -FVGK-------------DVLSDEILEVVCKYT---P--TPSS----------------  64
TbICP          -LAG--------------TELSDCTFAIQSKFNNRAPHDNHKNH---------------  70
Chagasin       -GTK--------------ESPNESMFTVENKYF---P---PDS----------------  62
EhICP-1        -CEG----------------LSNTVEYVADQHA---P----------------------  55
EhICP-2        -PTD---------------TFSLSQDTIKAEPH---P----------------------  74

PbICP          -----------------------------SKPKKPQMQLLGGPDRMRSVIK 321
PyICP          -----------------------------SKPKKPQMHLLGGPDNMRSVIK 324
Falstatin      -----------------------------HFPK-PREQLVGGSSMLISKIK 379
Toxostatin-1   --------------------------------------MPGAPQTYAAPVE 184
Toxostatin-2   VLFLKVFAEPTKKHGVVVSSPEKSMRSTPSTVETARGKMRRESY-YESEIS 225
LmICP          ----------------------------------TPMVGVGGIY--VVLVK  79
TbICP          ---------------------------------RRLLVGAGGT--MVLEVK  86
Chagasin       -----------------------------------KLLGAGGT--EHFHVT  76
EhICP-1        -----------------------------------GICGCGGKY--HIKIT  69
EhICP-2        ------------------------------------SGMVGFPSIREIQLK  89

PbICP          GHKPGKYYIVYSYYRPFSPTSGANTKIIYVTVQ- 354
PyICP          GHKAGKYYIVYSYYRPFSPTSGANTKILYVTVQ- 357
Falstatin      PHKPGKYFIVYSYYRPFDPTRDTNTRIVELNVQ- 412
Toxostatin-1   VTQVGDYTVIVSSVRPWAPRDAAGYYVVHIHATD 218
Toxostatin-2   SEVPGDFAVAFALVRPWKLSDQPQVFVALVHFD- 258
LmICP          PRKRGHHTLELVYTRPFEGIKPENERYTLHLNVK- 113
TbICP          ALKAGKHTLSLAYGRPWVGFNAAAKRYNIHVEATA 121
Chagasin       VKAAGTHAVNLTYMRPWTGPSHDSERFTVYLKAN- 110
EhICP-1        GTQTGEGKIVLVYRRPWAPN-ANDRTFTLKVNVQ- 102
EhICP-2        PLKVGTTTIKLGYSRPWEKGKEPLRSLTYSVVIR- 123
```
