# Supplementary figures and images for: Exoerythrocytic Plasmodium Parasites Secrete a Cysteine Protease Inhibitor Involved in Sporozoite Invasion and Capable of Blocking Cell Death of Host Hepatocytes
Source: PLoS Pathog. 2010 Mar 26;6(3):e1000825. doi: 10.1371/journal.ppat.1000825 (PMC2845656; doi:10.1371/journal.ppat.1000825)

A

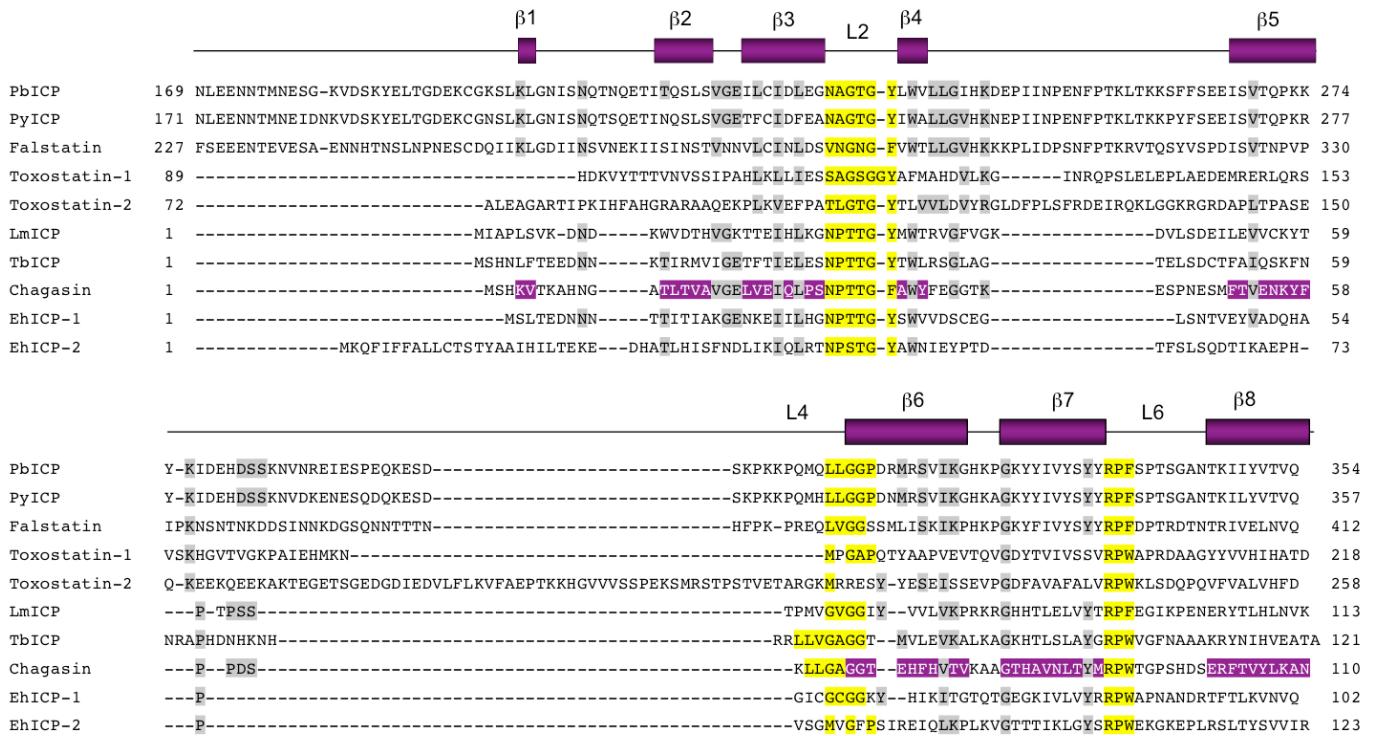

B

### Chagasin (*T. cruzi*) 12 kDa

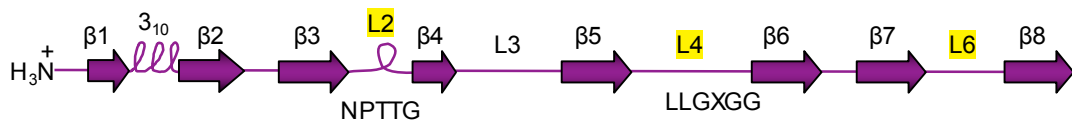

### PbICP (*P. berghei*) 40 kDa

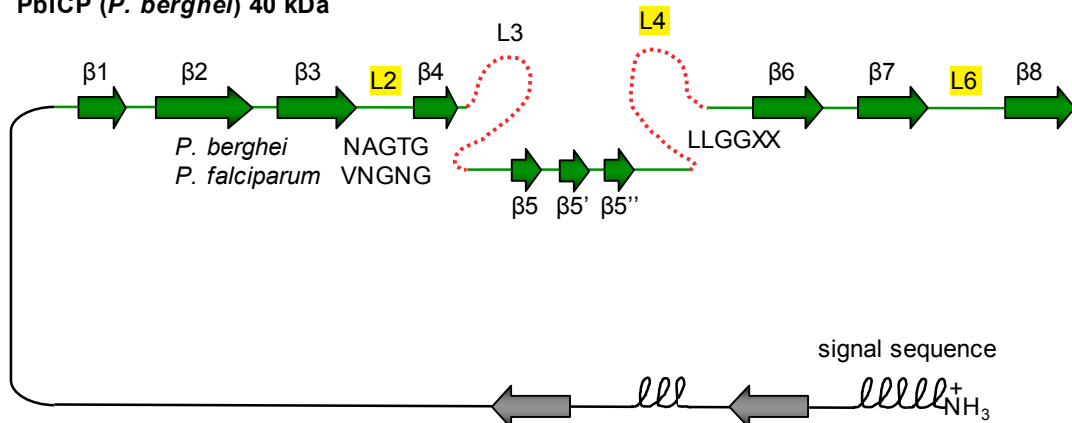

Supplement: Figure S2 — Multiple sequence alignment of the ICP chagasin domains. (A) Multiple sequence alignment of the C-terminal chagasin-like domain of PbICP, PyICP and falstatin/PfICP in comparison with the ICPs of T. gondii (toxostatins), T. cruzi (chagasin), T. brucei, L. mexicana and the two ICPs of E. histolytica. Conserved residues of the chagasin inhibitor family are highlighted in grey, the wedge forming loops that bind the active-site cleft of proteases (L2, L4, L6) are highlighted in yellow. At the top of the alignment the β-strands of chagasin are displayed in purple. The amino acid sequences of chagasin that form β-strands are additionally indicated in purple. (B) Known β-strands of chagasin and predicted β-strands of PbICP are depicted by arrows. In contrast to chagasin (purple), the inhibitor domain of PbICP (green) is predicted to have two additional β-strands (β5′ and β5′′) and elongated loop-structures L3 and L4 (red, dashed line) as well as a N-terminal extension region with a classic N-terminal signal sequence (grey). The wedge-forming loops that bind into the active site cleft of the proteases (L2, L4, L6) are highlighted in yellow. Like the toxostatins of T. gondii, but in contrast to the non-apicomplexan chagasin-like inhibitors of other protozoa and bacteria, the Plasmodium ICPs do not contain the NPTTG motif in L2 (variable motifs of P. berghei and P. falciparum are shown). (0.31 MB PDF) [file ppat.1000825.s002.pdf]

A

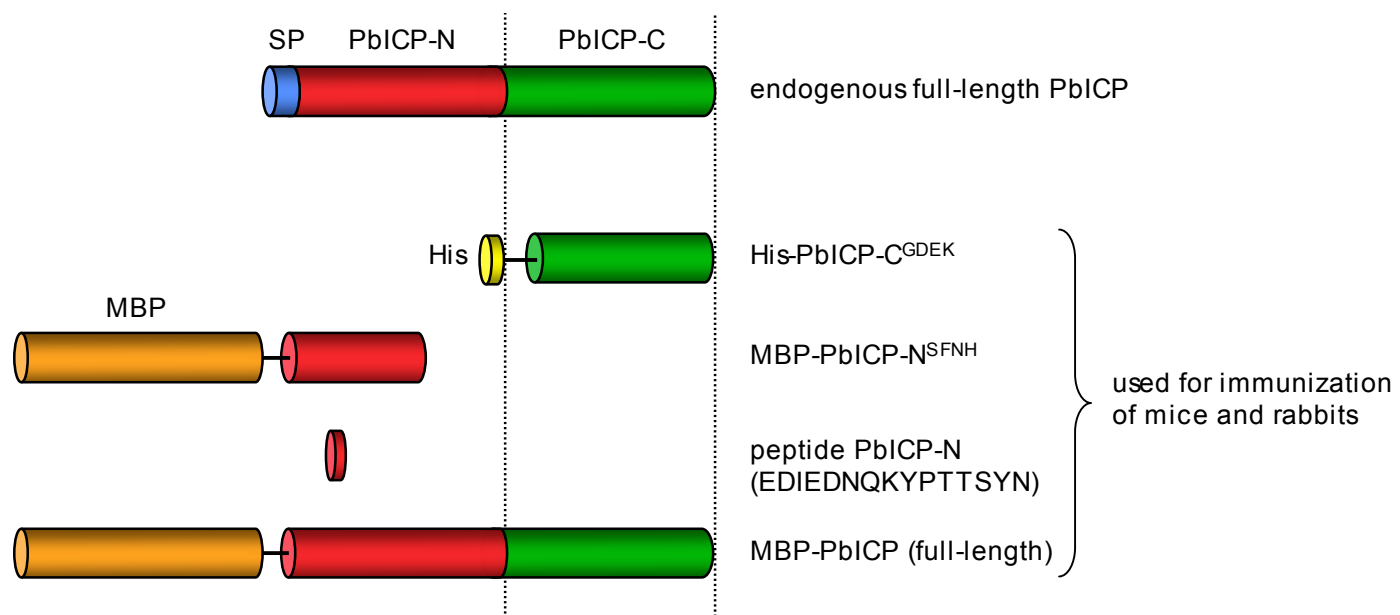

B

preimmune serum

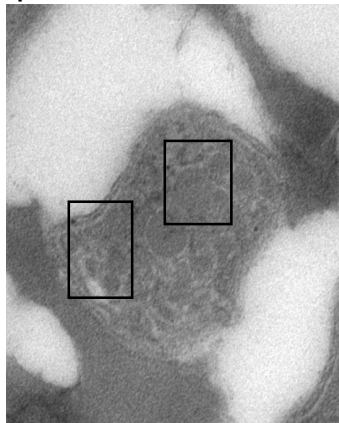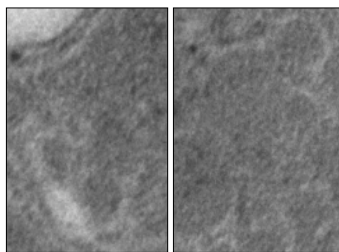

C

anti-His-PbICP-C<sup>GDEK</sup>

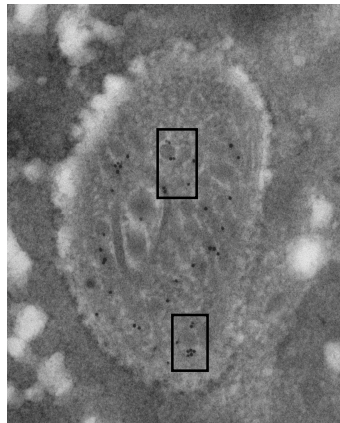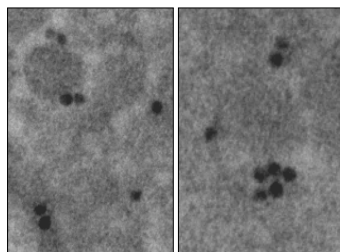

Supplement: Figure S3 — Schematic overview of PbICP constructs and epitopes used for the generation of antisera and examples of specificity controls. (A) To generate a specific antisera different regions of PbICP were used to immunize mice and rabbits. A mouse anti-PbICP antiserum was generated using MBP-tagged full-length PbICP. Recombinant His-PbICP-CGDEK was used for immunization to produce PbICP-C domain-specific antisera. Anti-PbICP-N domain-specific antisera were obtained from mice using recombinant MBP-PbICP-NSFNH for immunization and from rabbits using the peptide EDIEDNQKYPTTSYN. Panels (B and C) show a specificity control of the anti-PbICP-C antiserum (rabbit) in IEM. (7.94 MB PDF) [file ppat.1000825.s003.pdf]

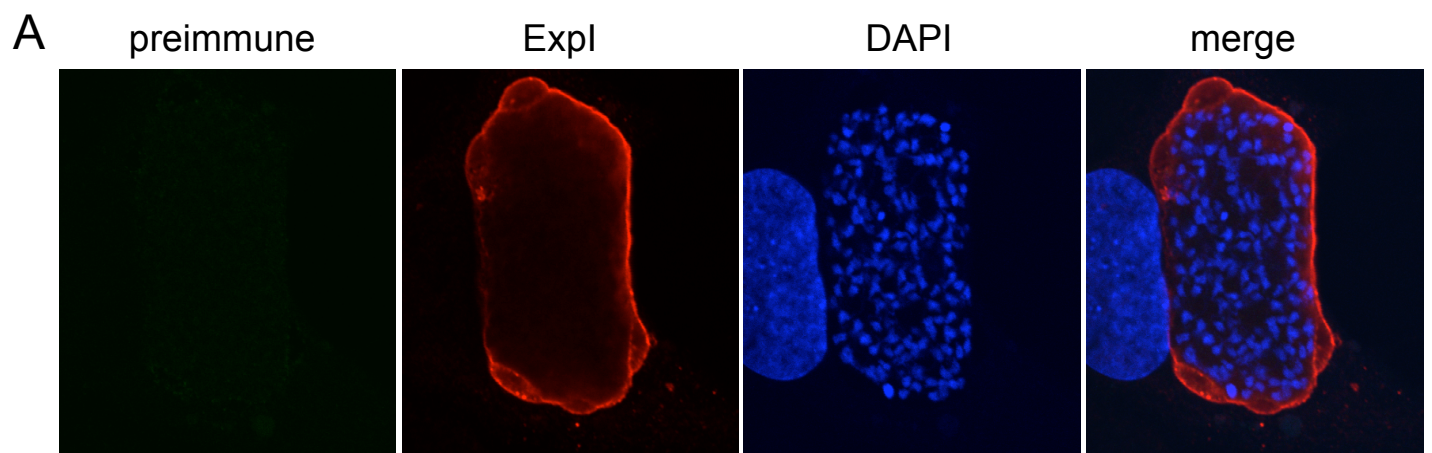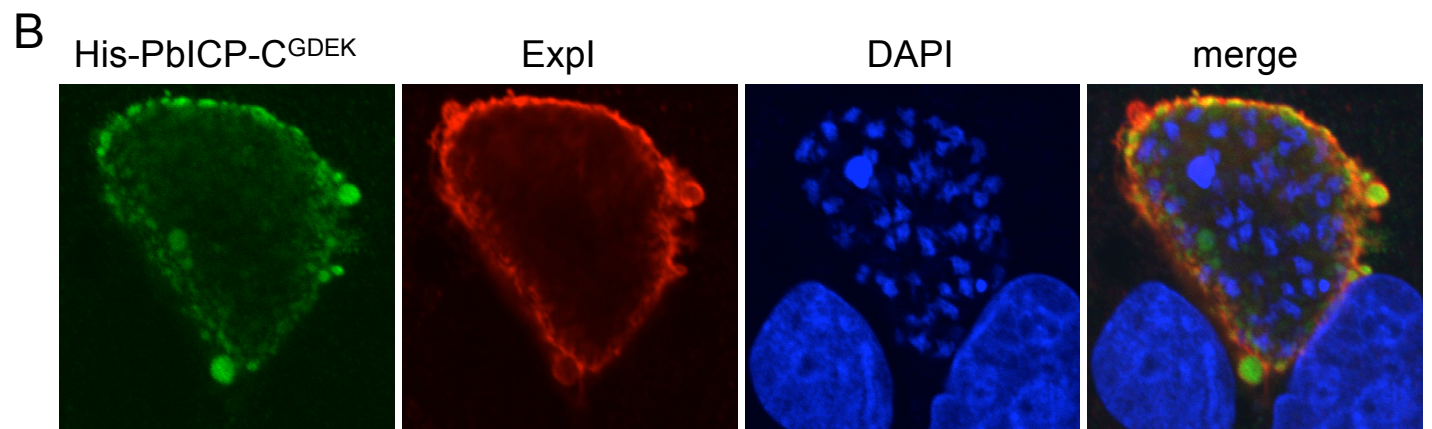

Supplement: Figure S4 — Specificity test of anti-PbICP-antiserum directed against His-PbICP-CGDEK. Confocal images of HepG2 cells infected with P. berghei wildtype parasites 55 hpi. Preimmune control (A) and anti-PbICP-C (B). Infected cells were fixed, incubated with a chicken anti-ExpI antiserum (secondary antibody: anti-chicken Alexa 594) and a rabbit antiserum against PbICP-C (secondary antibody: anti-rabbit Cy2) (B) or preimmune serum (secondary antibody: anti-rabbit Cy2) (A). DNA was stained with DAPI (blue). (1.67 MB PDF) [file ppat.1000825.s004.pdf]

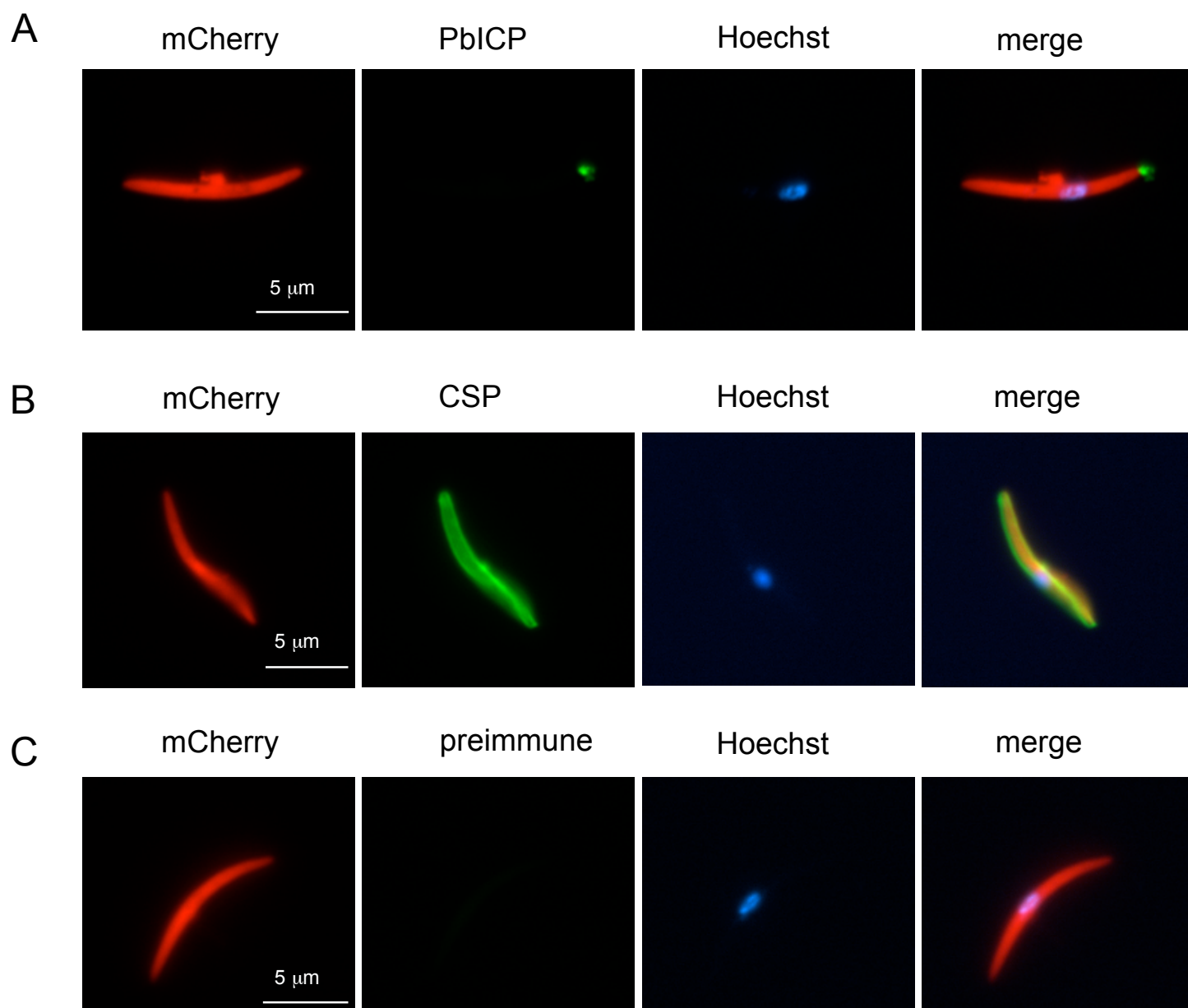

Supplement: Figure S5 — Staining of unfixed sporozoites shows PbICP localization at the apical pole of the sporozoite. Salivary gland sporozoites expressing mCherry were incubated on ice with rabbit anti-PbICP-C antiserum (A), rabbit anti-CSP antiserum (B) or rabbit preimmune serum (C), washed, subsequently stained with Cy2-conjugated secondary anti-rabbit antibody (green) and Hoechst 33258 (blue), again washed and immediately analyzed by fluorescence microscopy. (0.48 MB PDF) [file ppat.1000825.s005.pdf]

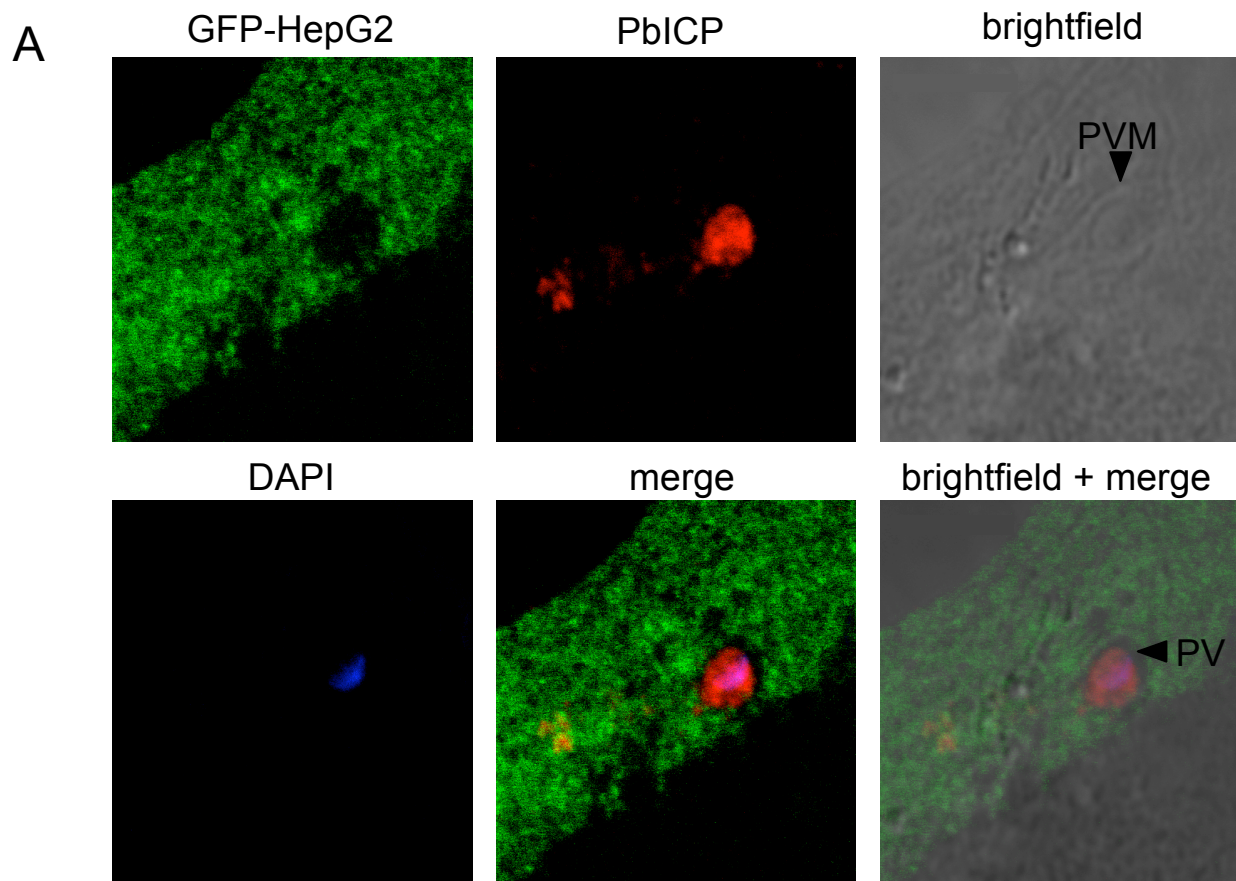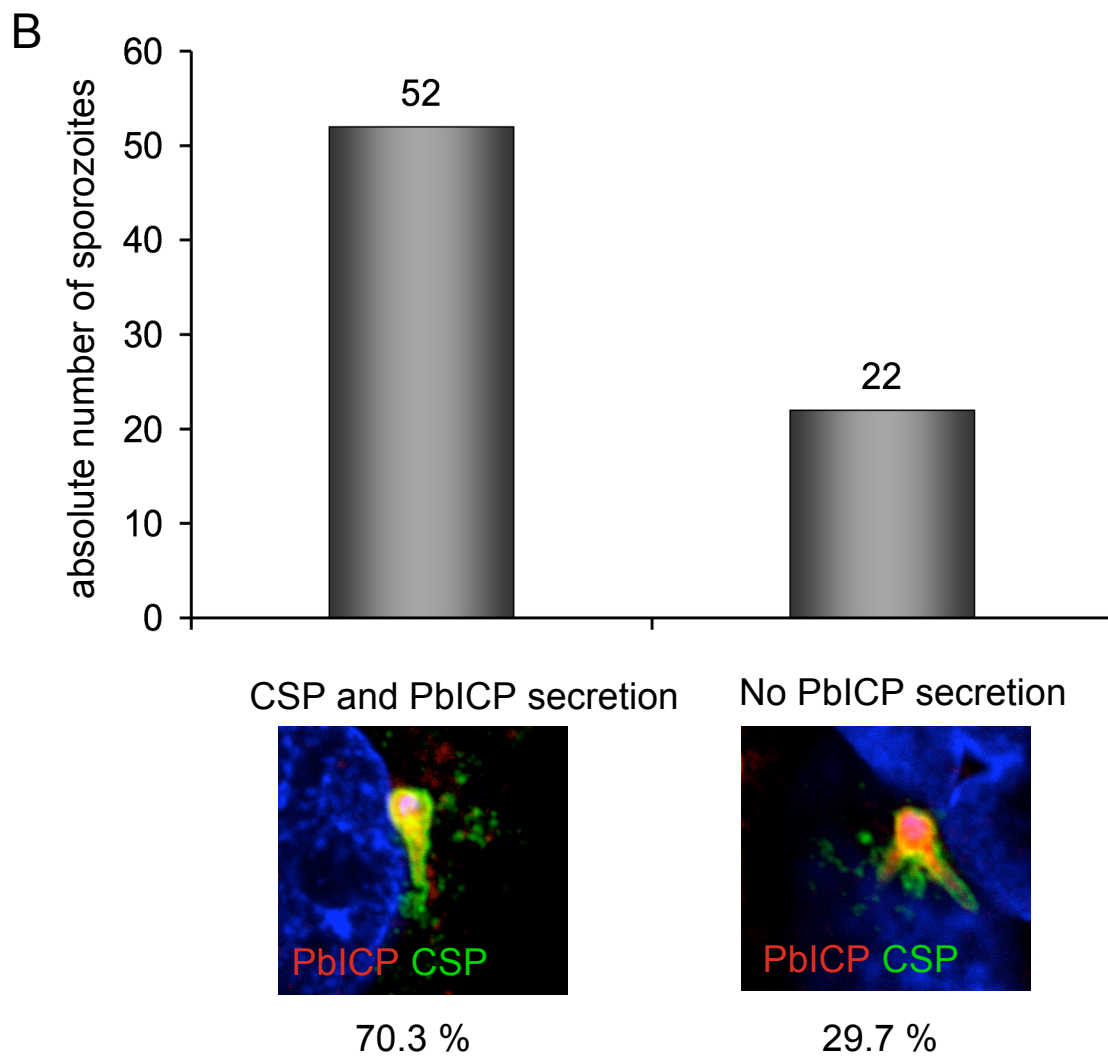

Supplement: Figure S6 — PbICP is secreted by intracellular trophozoites (confocal IFA). (A) IFA of a GFP-expressing HepG2 cell infected with P. berghei. Infected cells were fixed 4 hpi, incubated with polyclonal antisera against PbICP-C (rabbit) and against GFP (mouse) and subsequently stained with fluorescently labeled secondary antibodies (anti-rabbit, red and anti-mouse, green). DNA was stained with DAPI (blue). Partial co-localization of PbICP and GFP confirmed secretion of the inhibitor in the host cell cytoplasm. (B) Quantitative analysis of PbICP secretion. IFA of a HepG2 cell infected with P. berghei. Infected cells were fixed 4 hpi, incubated with polyclonal antiserum against PbICP-C (rabbit) and against CSP (mouse) and subsequently stained with fluorescently labeled secondary antibody (anti-rabbit conjugated with Cy2, green and anti-mouse conjugated with Alexa594, red). DNA was stained with DAPI (blue). Parasites associated with HepG2 cells and found in the same focal plane as the host cell nucleus were considered intracellular (see typical confocal images in inserts). Intracellular parasites were counted and the absolute numbers of parasites secreting either PbICP and CSP or CSP alone are shown in the graph. (1.76 MB PDF) [file ppat.1000825.s006.pdf]

PbICP

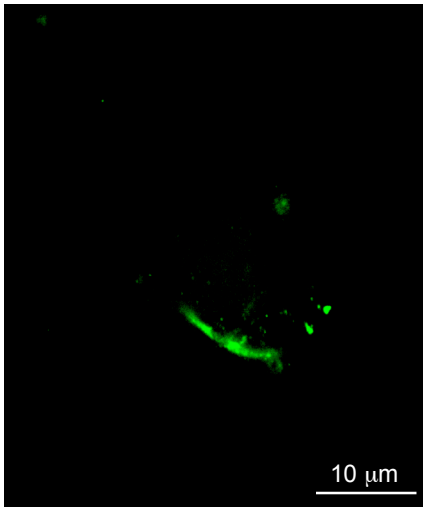

mCherry

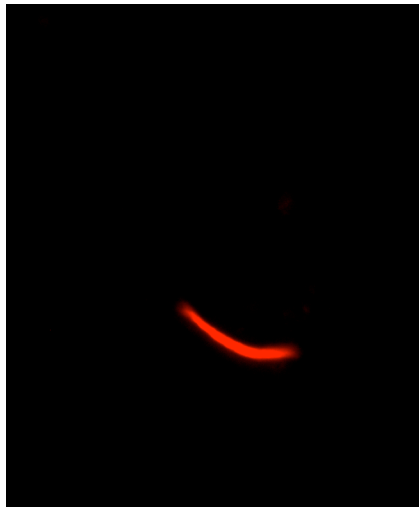

DAPI

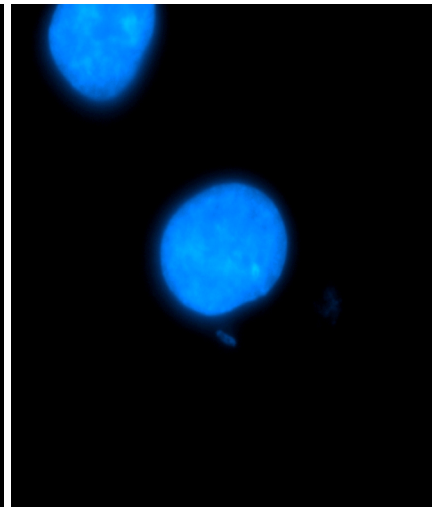

merge

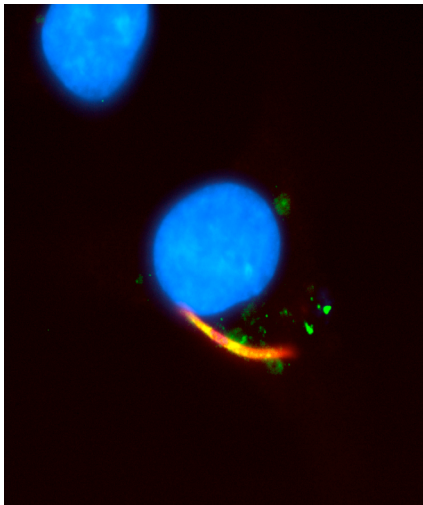

brightfield

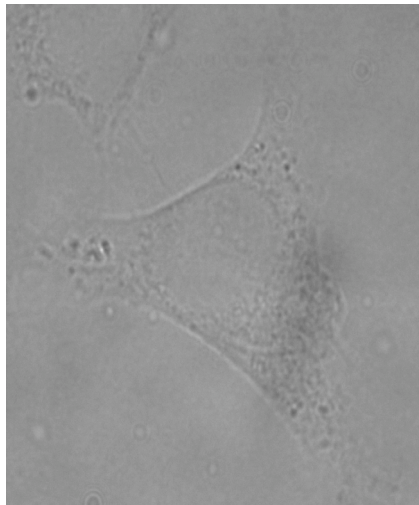

brightfield and merge

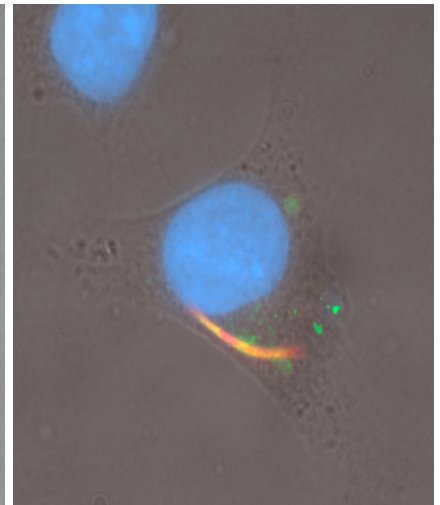

Supplement: Figure S7 — PbICP is secreted by intracellular sporozoites (widefield IFA). IFA of HepG2 cells infected with P. berghei (cytosolic mCherry expression, red) 2 hours after infection. Infected cells were fixed, incubated with polyclonal antiserum against PbICP-C (rabbit) and subsequently stained with fluorescently labeled secondary antibody (anti-rabbit conjugated with Cy2, green). DNA was stained with DAPI (blue). (1.61 MB PDF) [file ppat.1000825.s007.pdf]

PbICP

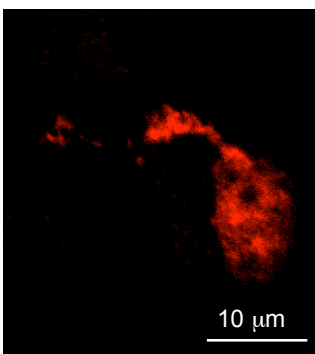

Expi

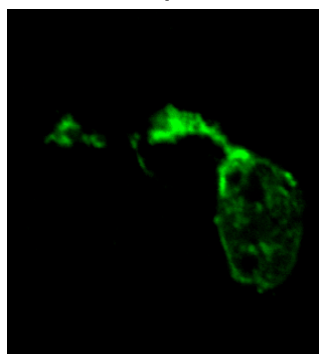

DAPI

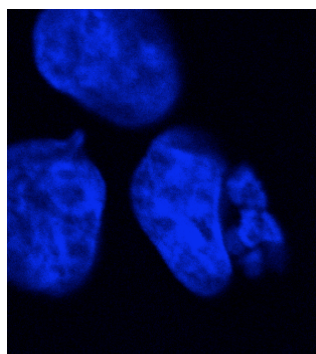

merge

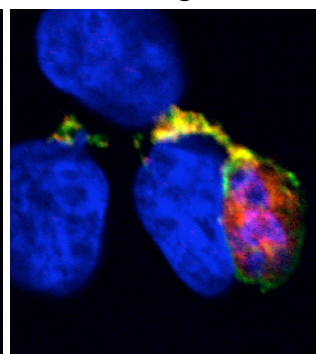

Supplement: Figure S8 — PbICP partially co-localizes with the PVM marker ExpI at the schizont stage (confocal IFA). Confocal IFA of P. berghei-infected HepG2 cells. Cells were fixed 30 hpi and stained with polyclonal antisera against PbICP-C (rabbit, red) and ExpI (chicken, green). DNA was stained with DAPI (blue). (0.35 MB PDF) [file ppat.1000825.s008.pdf]

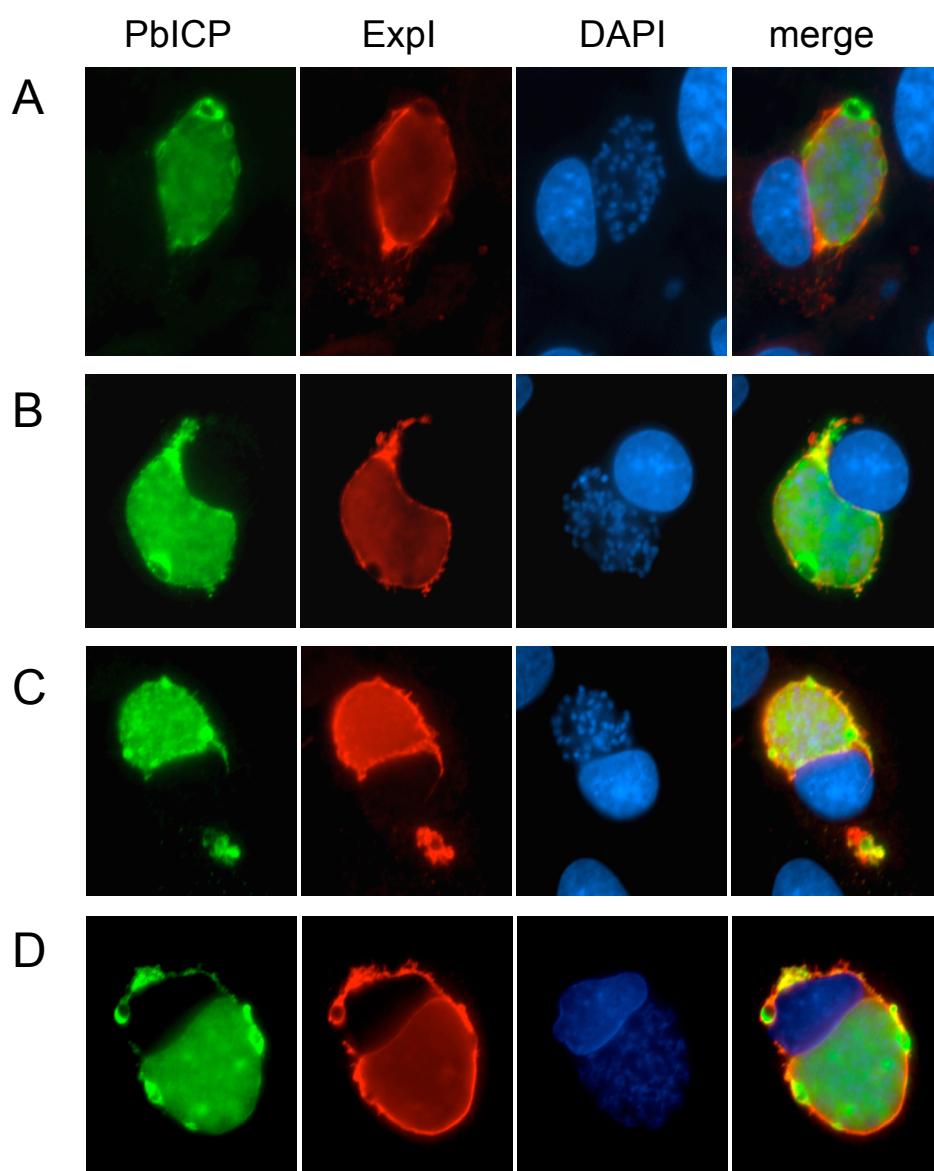

Supplement: Figure S9 — PbICP localizes to vesicular structures in the PV of liver stage schizonts. IFA of HepG2 cells infected with P. berghei at 48 hpi. Infected cells were fixed and stained with anti-ExpI antiserum (chicken, red) and polyclonal antiserum against PbICP-C (rabbit, green). DNA was stained with DAPI (blue). Representative images are presented in A-D. (2.00 MB PDF) [file ppat.1000825.s009.pdf]

frequency of the phenotype

**47.9 ± 14.1 %**

**0.9 ± 1.5 %**

**2.6 ± 2.2 %**

**3.7 ± 1.8 %**

**44.9 ± 14.9 %**

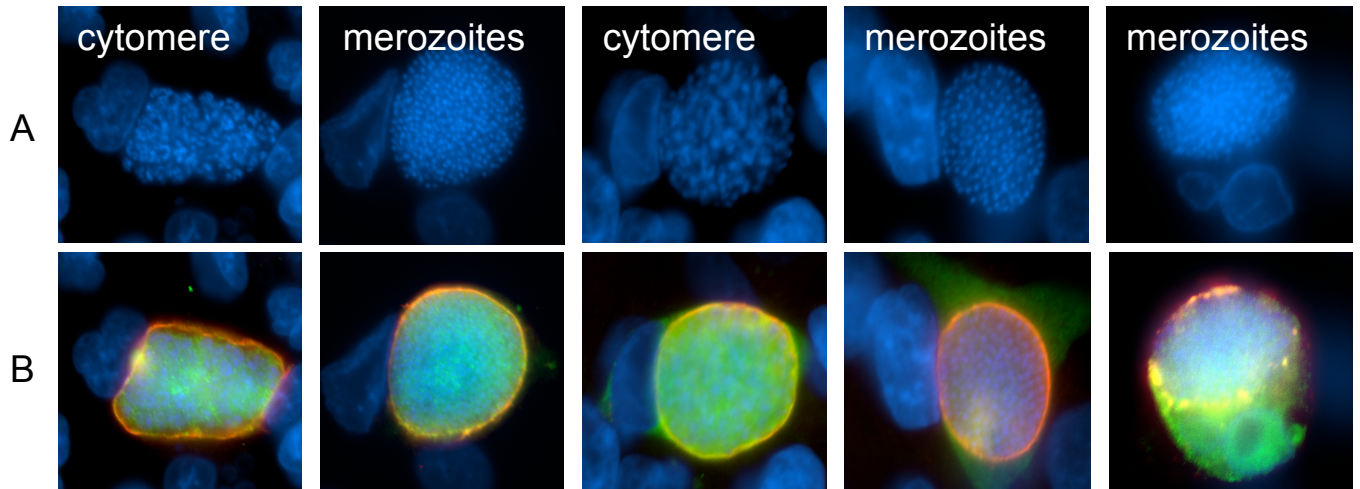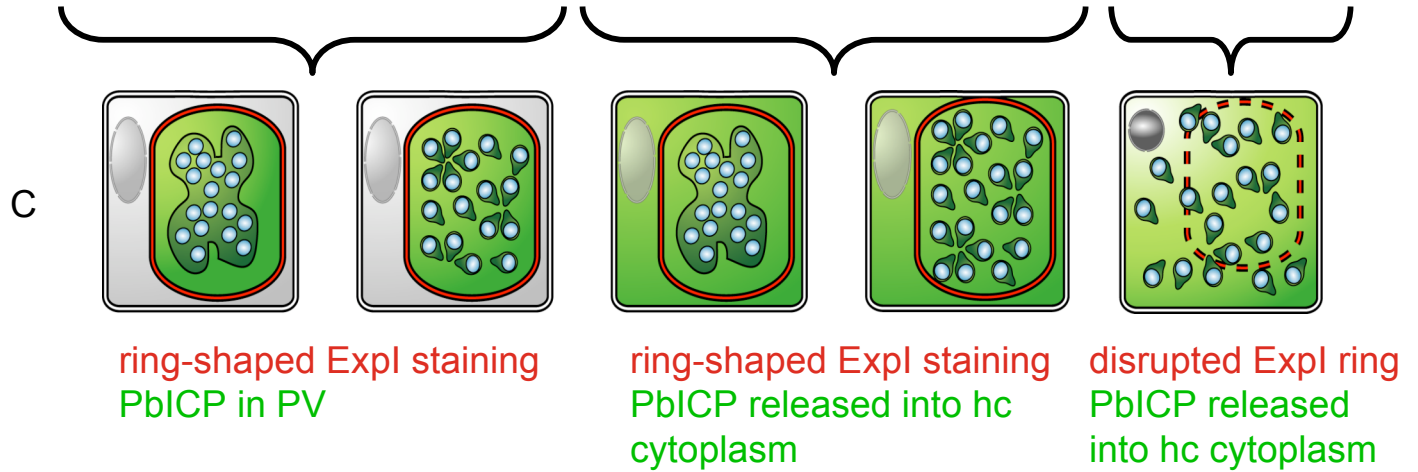

Supplement: Figure S10 — PbICP is released into the host cell cytoplasm at the end of the liver stage. IFA of HepG2 cells infected with P. berghei at the end of the liver stage (63 hpi) prior to and after visible destruction of the PVM. Infected cells were fixed, stained with DAPI (A) and with anti-ExpI antiserum (chicken, red) and polyclonal antiserum against PbICP-C (mouse, green) (B). Different phenotypes are presented as a cartoon (C). Late schizont/merozoite stages were counted and the percentage of each different phenotype was calculated. Presented on top of the images are the means and standard deviations of three independent experiments (frequency of phenotypes). Main phenotypes are parasites with intact PVM and PbICP restricted to the parasite and the PV, and parasites with disrupted PVM visible by Exp1 staining and PbICP release into host cell cytoplasm. hc: host cell. (3.02 MB PDF) [file ppat.1000825.s010.pdf]

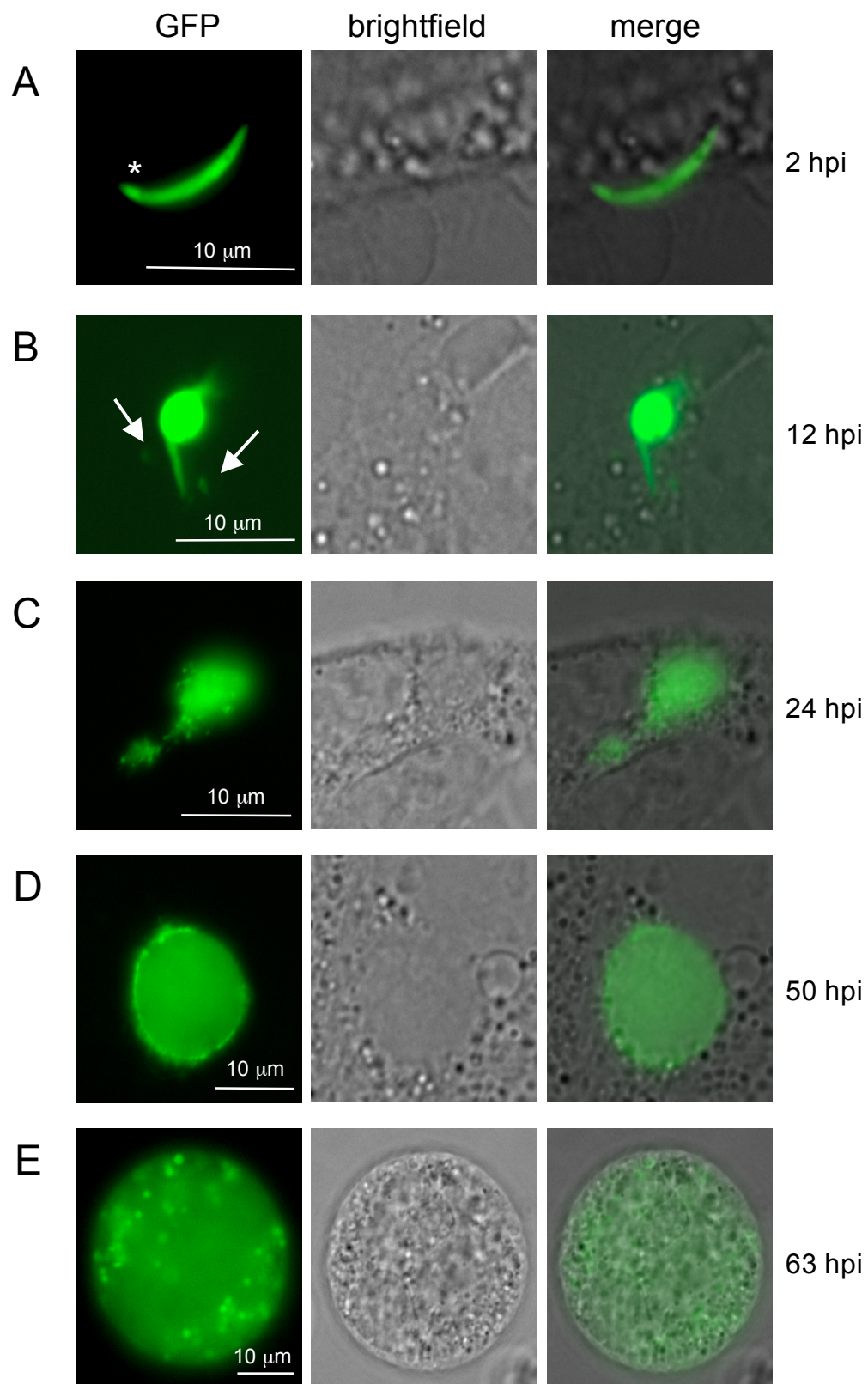

Supplement: Figure S11 — Characterization of the PbICP-GFP-expressing liver stage parasites. (A-E) Live imaging of PbICP-GFP-expressing liver stage parasites confirmed the PbICP localization determined by the antisera-based analysis. HepG2 cells were incubated with PbICP-GFP-expressing P. berghei parasites and analyzed at different time points after infection. The sporozoite shown in panel (A) revealed an apical accumulation of the GFP fluorescence (marked with an asterisk). Early liver stage parasites (B) released GFP-positive structures (marked with arrows). In schizont stages (C, D), GFP fluorescence was found in the PV and the parasite cytosol. At the end of the liver stage, after detachment of the infected HepG2 cell (E), GFP fluorescence was found in the host cell cytoplasm and in the merozoites. (3.78 MB PDF) [file ppat.1000825.s011.pdf]

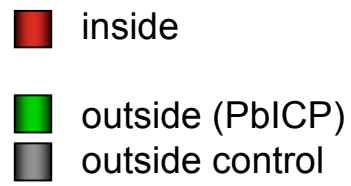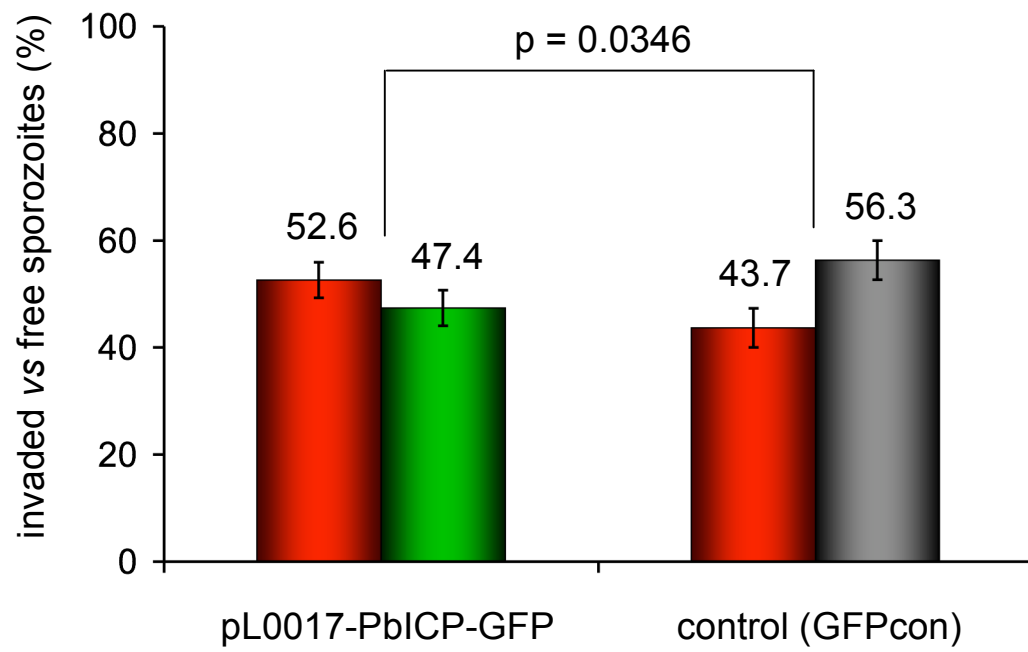

Supplement: Figure S12 — PbICP-GFP-expressing P. berghei show slightly enhanced infection efficiency. HepG2 cells were infected with transgenic PbICP-GFP sporozoites or GFPcon sporozoites as a control, incubated for 1 h, subsequently fixed without permeabilization and stained with an anti-CSP antiserum (inside/outside assay). Extracellular but not intracellular sporozoites were labeled by the anti-CSP antiserum. Intracellular sporozoites are only positive for GFP expression. Sporozoites were counted and the percentages of free and intracellular sporozoites were calculated. Presented are the means and standard deviations of three independent experiments. (0.09 MB PDF) [file ppat.1000825.s012.pdf]

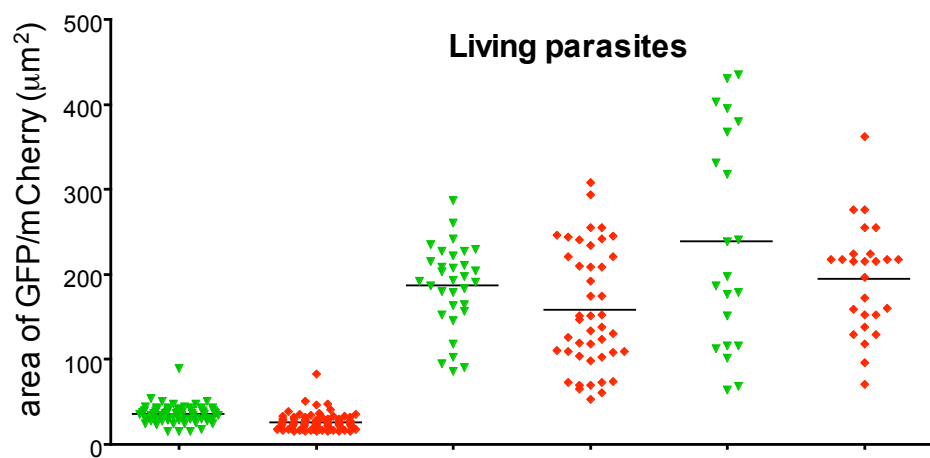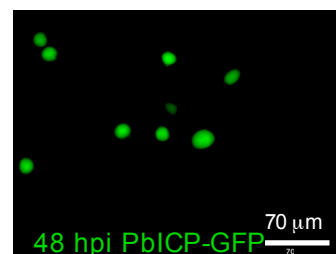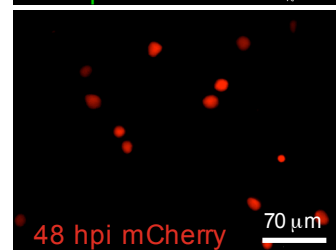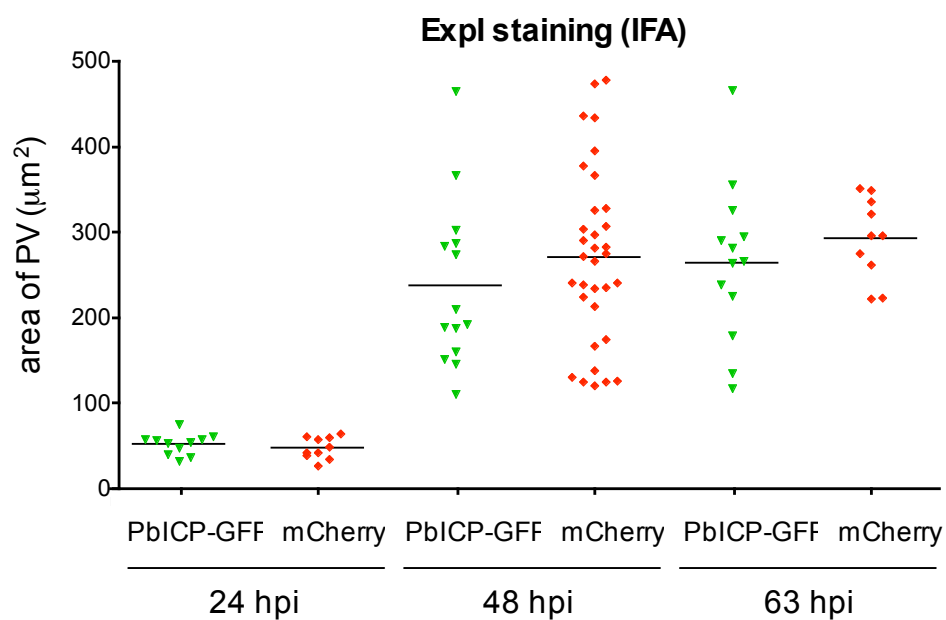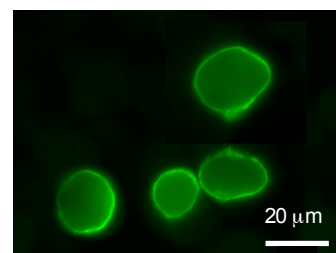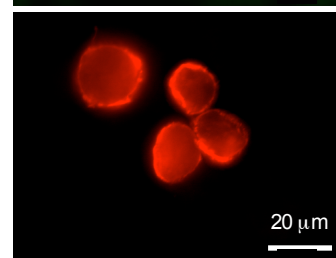

Supplement: Figure S13 — PbICP-GFP expressing parasites do not differ in their intrahepatic development from mCherry-expressing parasites. HepG2 cells were infected with transgenic PbICP-GFP sporozoites or mCherry-expressing sporozoites. Parasite size was determined over the course of development in HepG2 cells using the density slice module of the OpenLab 5.03 software. Size was measured at 24, 48 and 63 hpi by live imaging. Since mCherry expression is restricted to the parasite cytosol but PbICP-GFP is also translocated into the PV, PbICP-GFP-expressing parasites appear slightly bigger (upper panel). To analyze this observation in more detail, infected cells were fixed and stained with an anti-Exp1 antiserum, which labels the PVM of both parasite strains (lower panel). In contrast to the live imaging, this experiment revealed that mCherry parasites are slightly bigger confirming that PbICP-GFP is secreted into the PV. (0.38 MB PDF) [file ppat.1000825.s013.pdf]

A

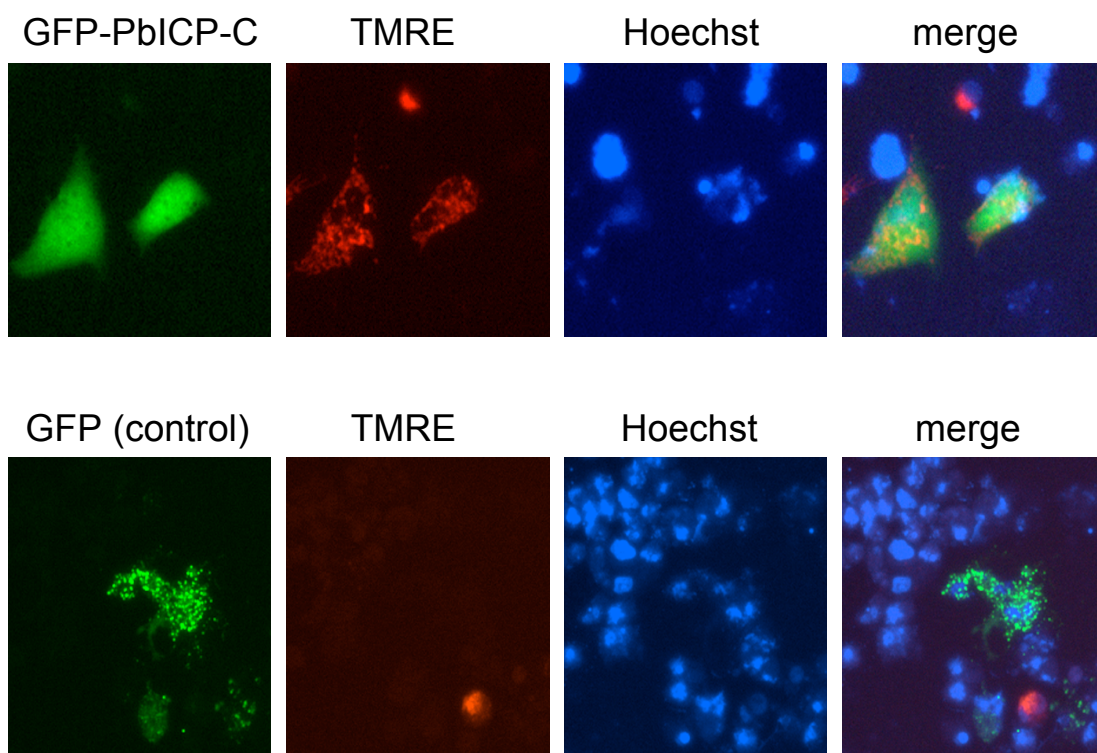

B

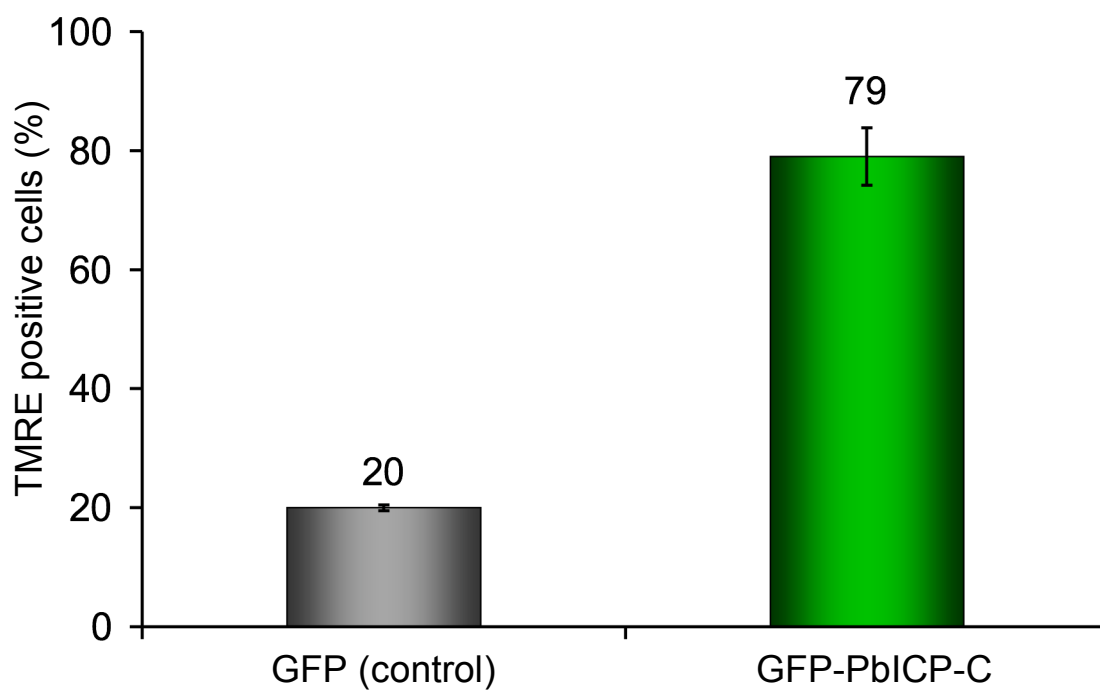

Supplement: Figure S14 — PbICP-C expression protects HepG2 cells against host cell death (camptothecin treatment). (A) HepG2 cells were transiently transfected with a plasmid leading to cytosolic expression of GFP-tagged PbICP-C (upper panel) or with a GFP control plasmid (lower panel). Subsequently host cell death was induced by camptothecin treatment for 24 h and analyzed by live imaging of intact mitochondria by TMRE (red) and DNA by Hoechst (blue). Dying cells exhibited condensed chromatin in the nucleus and a loss of mitochondrial membrane potential. (B) Fluorescent cells were counted and the percentages of dead and viable cells were calculated. Cells expressing GFP-PbICP-C showed significantly better survival upon camptothecin-induced cell death in comparison to GFP-expressing cells. (1.28 MB PDF) [file ppat.1000825.s014.pdf]
